# Fluorescent proteins generate a genetic color polymorphism and counteract oxidative stress in intertidal sea anemones

**DOI:** 10.1073/pnas.2317017121

**Published:** 2024-03-08

**Authors:** D. Nathaniel Clarke, Noah H. Rose, Evelien De Meulenaere, Benyamin Rosental, John S. Pearse, Vicki Buchsbaum Pearse, Dimitri D. Deheyn

**Affiliations:** ^a^Department of Biology, Hopkins Marine Station, Stanford University, Pacific Grove, CA 93950; ^b^Marine Biology Research Division, Scripps Institution of Oceanography, University of California, San Diego, La Jolla, CA 92037; ^c^The Shraga Segal Department of Microbiology, Immunology and Genetics, Faculty of Health Sciences, Center for Regenerative Medicine and Stem Cells, Ben-Gurion University of the Negev, Beer-Sheva 84105, Israel; ^d^Department of Ecology and Evolutionary Biology, Joseph M. Long Marine Laboratory, University of California, Santa Cruz, CA 95060

**Keywords:** GFP, polymorphism, antioxidant, biofluorescence, *Anthopleura*

## Abstract

Fluorescent proteins (FPs) are ubiquitous tools in research, yet their endogenous functions in nature are poorly understood. In this work, we describe a combination of functions for FPs in a clade of intertidal sea anemones whose FPs control a genetic color polymorphism together with the ability to combat oxidative stress. Focusing on the underlying genetics of a fluorescent green “Neon” color morph, we show that allelic differences in a single FP gene generate its strong and vibrant color, by increasing both molecular brightness and FP gene expression level. Natural variation in FP sequences also produces differences in antioxidant capacity. We demonstrate that these FPs are strong antioxidants that can protect live cells against oxidative stress. Finally, based on structural modeling of the responsible amino acids, we propose a model for FP antioxidant function that is driven by molecular surface charge. Together, our findings shed light on the multifaceted functions that can co-occur within a single FP and provide a framework for studying the evolution of fluorescence as it balances spectral and physiological functions in nature.

## Significance

Fluorescent proteins (FPs) revolutionized molecular biology, yet their natural functions remain largely mysterious. Our study illuminates roles for FPs in sea anemones, demonstrating their ability to control color variations while also protecting against oxidative stress. Remarkably, allelic differences in just one FP gene govern a vibrant color polymorphism. Further, we show how the same FPs shield against oxidative damage, offering a model for FP antioxidant action. Our insights underscore an intriguing evolutionary balance between the chromatic and physiological roles of FPs. This broadens our understanding of animal adaptation to environmental challenges and offers potential refinements in the application of FPs in scientific research.

Since the discovery of Green Fluorescent Protein (GFP) in the jellyfish *Aequorea victoria* (1), FPs have become indispensable tools in cellular and molecular biology (2). However, despite six decades of extensive research and engineering, the native functions of FPs in the cells and tissues of cnidarians, and the evolutionary processes driving their origin and diversification, remain largely unknown.

To explore FP functional evolution, comparative studies have described the unique functions of FPs and delineated their distribution across the animal kingdom and across environments. FPs are found in a diverse array of animals, primarily in cnidarians (3, 4) and also in some crustaceans (5, 6) and cephalochordates (7–9). Researchers have proposed a myriad of functions, ranging from visually signaling prey or predators (10, 11) to physiology (12–14). Anthozoans, such as corals and sea anemones, have the largest diversity of FPs (3, 15, 16), with roles linked to photobiology, such as the regulation of algal endosymbiosis (17, 18) and protection against ultraviolet light radiation (12), or to physiology, such as antioxidant activity (13). To what extent these functional properties co-occur, or are mutually exclusive, is largely unknown. However, a common assumption is that FPs may have evolved from physiologically active chromoproteins (CPs) and were secondarily co-opted in some organisms to perform chromatic functions, which in turn would suggest that these roles are mutually exclusive (15). To this day, this has remained speculative and still open to debate.

It is well recognized that comparative studies across animals and environments, while insightful, often fall short of elucidating the sequence-level or structural features acted upon by natural selection to optimize for specific functions, thus leaving a crucial gap in our understanding of FP molecular evolution. To bridge this gap and explore the evolutionary trajectory of FP functions, we must identify organisms utilizing FPs for multiple purposes and study how sequence variation in FP genes affects their functionality. We used this approach by examining the mechanistic basis of FP function in three intertidal sea anemones of the genus *Anthopleura*. These animals offer a unique opportunity to scrutinize the evolution and diversification of FPs under both photobiological and physiological selective pressures, because species of *Anthopleura* display natural color variation within and between species (19, 20) and inhabit physiologically stressful intertidal habitats (21–23).

On the Pacific coast of North America, three closely related species of *Anthopleura* reside in rocky intertidal habitats: *Anthopleura elegantissima*, *Anthopleura sola*, and *Anthopleura xanthogrammica* (24, 25). Each species occupies a preferred microhabitat niche and has unique lifestyle and habits, but all face a similar barrage of stressors from their environment. The rocky intertidal zone is a dynamic environment where organisms face gradients of stresses: exposure to desiccation, ultraviolet radiation, temperature extremes, and salinity fluctuations varies spatially from high to low tide zones, and temporally from daily tidal cycles to seasonal climatic shifts (26). Among other adaptations, these sea anemones harbor algal endosymbionts and subsist as photoautotrophs under optimal light conditions, or as heterotrophs feeding on prey and detritus as necessary (24, 27). Their color varies, apparently based on a combination of environmental and genetic factors, in addition to the abundance and composition of the endosymbiont microbiome (28–30).

In this study, we characterize multiple variants of AnthoYFPs, a sub-family of GFPs derived from these three species of *Anthopleura*. We show that allelic variants of AnthoYFPs control a color polymorphism in the Sunburst Anemone, *A. sola*. Investigating the underlying genetics across color morphs, we find that the “Neon” color phenotype is a trait exhibiting incomplete dominance; it correlates with copy number of a *Neon* AnthoYFP allele encoding higher levels of gene expression and a visibly brighter variant of AnthoYFP. Comparative biochemical analysis uncovers a physiological function for AnthoYFPs: they can act as strong antioxidants in vitro and in vivo. We also identify local variation in protein surface charge as a mechanism controlling antioxidant capacity. This suggests a broader physiological role for FPs in anthozoan cnidarians, acting as biochemical buffers against the numerous oxidative stressors occurring in the intertidal ecosystem. Overall, our findings demonstrate natural co-occurrence and variation of spectral and biochemical/physiological FP functions within anemone populations and suggest that FPs can play multiple roles in anemone biology.

## Results

### A Fluorescent Neon Color Polymorphism in the Sunburst Anemone, *A. sola*.

**Fig. 1.**
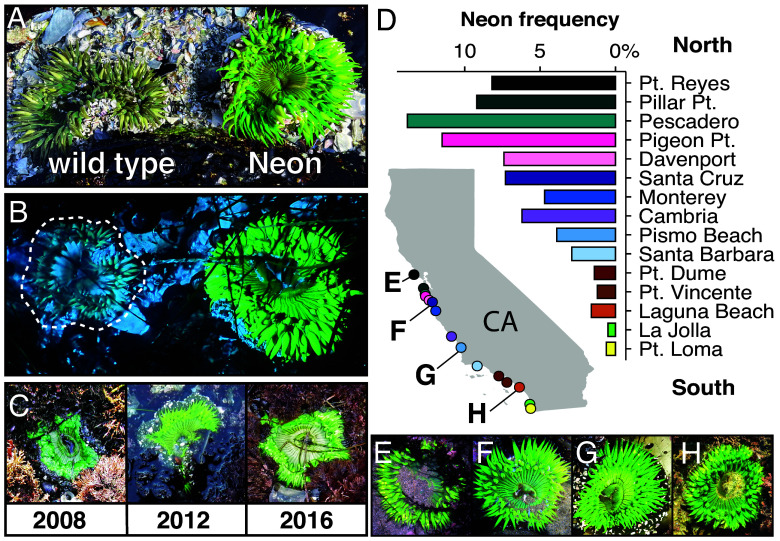
A striking Neon color morph of the Sunburst Anemone, *A. sola*, occurs at a low frequency in anemone populations in a latitudinal gradient along the California coast. (*A* and *B*) Comparison of wild type (*Left*) versus the vibrant Neon color morph (*Right*) under sunlight (*A*) or blue light illumination (*B*). (*C*) Photos of a single Neon individual in Pacific Grove, CA taken over multiple years, demonstrating the stability of the Neon phenotype over time. (*D*) Histogram of count frequencies along the CA coast from geotagged, research-grade observations of *A. sola* from the iNaturalist database. Bar colors correspond to map locations. (*E*–*H*) Images of Neon individuals from locations across the species range indicated in (*D*).

We identified a rare neon-green pigmentation phenotype in *A. sola* that contrasts sharply with the wild-type olive to gray-green varieties commonly seen intertidally in Central California. This striking Neon phenotype consists of an intense green pigmentation in the oral disk and tentacles that is conspicuous under natural sunlight (Fig. 1*A*) and exhibits bright yellow-green fluorescence under blue light (Fig. 1*B*). Because coloration in *Anthopleura* spp. can vary with multiple habitat factors, such as temperature and light exposure (28–30), it was unclear whether the Neon phenotype represented an ephemeral environmental response or a genetically encoded color morph. To address this, we examined the persistence of the Neon phenotype and surveyed its distribution along the California coast. Long-term monitoring from August 2004 to June 2023 revealed that the Neon phenotype was stably maintained in individuals over decadal time scales (Fig. 1*C*). Analysis of geo-tagged observations of *A. sola* from the iNaturalist community science database confirmed that the Neon phenotype is not restricted to central California (Fig. 1*D*). The Neon phenotype occurred at a low frequency (1.8%, n = 5,968 images) from Pt. Reyes, CA (38°N) to Isla del Cedros in Baja, Mexico (28°N) covering ~70% of the species range (*SI Appendix*, Fig. S1). Analysis of the geographic distribution of Neon anemones revealed a North-to-South cline in observation frequency, with the Neon morph most common in Central California south of San Francisco Bay (Pescadero, 13.8%), decreasing to near-zero around San Diego (Cabrillo Pt., 0.6%; 1 of 164 observations) (Fig. 1*D*).

### The Source of Green Pigmentation Is a Fluorescent Protein, AnthoYFP.

Green pigmentation in these species of *Anthopleura* is commonly attributed to the presence of algal endosymbionts (24, 31). However, the southern limit of green zoochlorellae is 38°N (Point Reyes), coinciding with the northern limit of *A. sola,* and only brown zooxanthellae occur as symbionts south of this latitude (32). This indicates that the anemones’ green color is not due directly to their algal symbionts; indeed, the abundant green pigmentation might be endogenously produced by the host anemone, wholly independently of the algal endosymbionts’ presence.

To determine which organism (host vs. symbiont) produces the green color of the anemones and confirm that anemone pigment is the source of bright fluorescence, we examined tentacles of *A. sola* by microscopy. Both Neon morph and wild-type exhibited green fluorescence in anemone cells on the oral (upper) side of the ectoderm, yet not in the symbiont-bearing endoderm (*SI Appendix*, Fig. S2 *A* and *B*). Area-normalized fluorescence intensities were ~4-fold greater in Neon tentacles compared to wild type (33.6 vs. 128.9 mean intensity; *P* = 0.0006, Wilcox test, n = 14; *SI Appendix*, Fig. S2*C*); no differences in the location of green fluorescence or in symbiont cell number were observed between morphs (*SI Appendix*, Fig. S2 *D* and *E*). Spectral measurements of tentacle lysates via Excitation Emission Matrix (EEM) revealed two EEM peaks in both morphs: one associated with the green pigment (515/530 nm) and the other with algal chlorophylls (430/675 nm) (*SI Appendix*, Fig. S3 *A*–*C*) (33, 34). Isolation of a pure green pigment by column chromatography indicated that this molecule is the sole source of the 530 nm peak (*SI Appendix*, Fig. S3 *D* and *E*). The 530-nm pigment was also found in lysates from symbiont-free wild-type anemones living in constant darkness, confirming that green pigment production does not depend on the presence of symbionts or environmental light exposure in *A. sola* (*SI Appendix*, Fig. S3 *C* and *F*).

Based on the location of the green fluorescence (*SI Appendix*, Fig. S2), its spectral dissimilarity with algal photopigments (*SI Appendix*, Fig. S4), and its production in aposymbiotic individuals, we inferred that its source is a FP produced solely by the anemone. SDS-PAGE analysis of the isolated pigment confirmed that it is a ~27-kDa yellow-green fluorescent protein with a peak absorption of 516 nm (Table 1). Size exclusion chromatography and multi-angle light scattering analysis demonstrated that it exists as an obligate tetramer with an apparent molecular weight of 111.1 kDa (*SI Appendix*, Fig. S5). We determined its amino acid sequence using Edman degradation sequencing and mass spectrometry (*SI Appendix*, Fig. S6), and its DNA coding sequence via Sanger sequencing of PCR products amplified from cDNA with degenerate primers. BLAST homology searches revealed that the protein, which we named *Anthopleura* YFP (AnthoYFP), is in the GFP family, and is most similar to cjFP510 from the deep sea anemone *Cribrinopsis japonica* (identity = 69.9%, E-value = 9e^−114^; BLASTP against non-redundant protein sequences), and asFP499 from *Anemonia sulcata* (identity = 66.8%, E-value = 1e^−110^) (35, 36).

**Table 1.** Spectral properties and antioxidant capacity of purified AnthoYFPs in comparison to EGFP and EYFP

	Abs. max (nm)	Ex. Max (nm)	Em. Max (nm)	Extinction coefficient (10^3^ M^−1^ cm^−1^)	Quantum yield	Theoretical brightness (EC * QY * 10^−3^)	Antioxidant^*^ capacity (µmol/µmol T.E.)
**EGFP**	488	488	507	55,900	0.6	33.54	0.40 ± 0.16*
**EYFP**	512	512	527	67,000	0.67	44.89	0.13 ± 0.11
**Neon *A. sola***	516	515	530	74,500	0.59	43.96	0.42 ± 0.07*
**Wild-type *A. sola***	514	514	531	70,800	0.26	18.41	0.20 ± 0.13*
***A. xanthogrammica***	512	514	529	35,300	0.32	11.31	1.96 ± 0.14*
***A. elegantissima***	514	514	528	49,200	0.24	11.80	0.35 ± 0.08*

^*^Antioxidant capacity is expressed as molar equivalent to the reference standard, Trolox (T.E. = Trolox equivalent), and shown as the mean ± SD, calculated from 10 replicate experiments. Proteins with antioxidant capacity significantly different than the buffer blank are noted with an asterisk (*).

The similar pigmentation of *A. sola*, *A. xanthogrammica,* and *A. elegantissima* suggested that AnthoYFP contributes to the visible green color of all three species. We found orthologous sequences in *A. xanthogrammica* and *A. elegantissima* and observed that both species emit green-yellow fluorescence under blue light (*SI Appendix*, Fig. S7). Phylogenetic analysis confirmed that AnthoYFPs are closely related orthologs within the larger anthozoan GFP clade (*SI Appendix*, Fig. S8).

### Neon Anemones Are Homozygous for a *Neon* AnthoYFP Allele that Correlates with Intensity of Green Pigmentation.

**Fig. 2.**
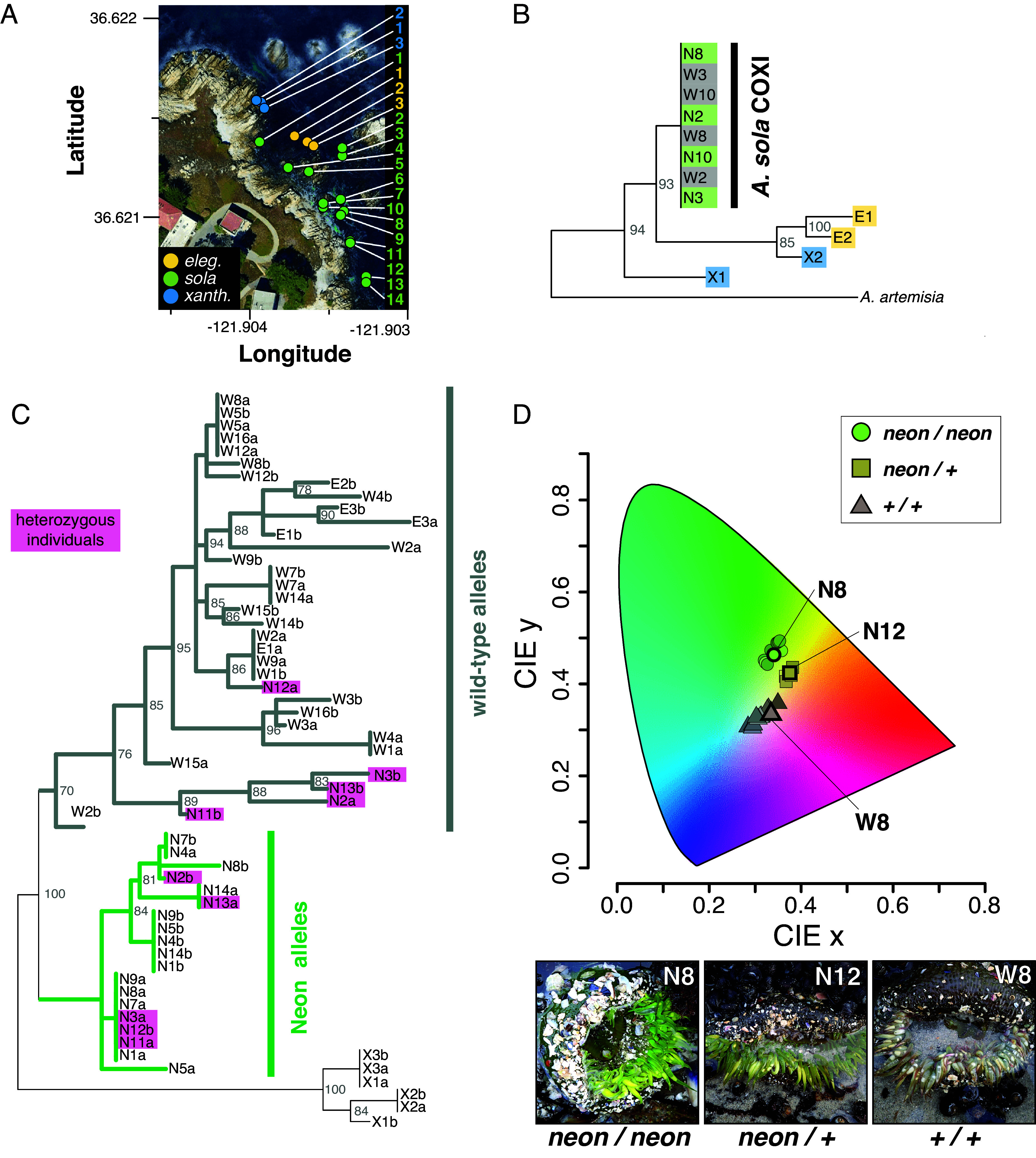
Neon anemones are homozygous for a low-frequency AnthoYFP allele that correlates with the level of green pigmentation. (*A*) Sampling location of anemones in the intertidal at Hopkins Marine Station used in genetic analyses (*B* and *C*). Positions shown for *A. sola* are of Neon anemones; paired WT samples were collected from the nearest adjacent WT neighbor. (*B*) Maximum likelihood tree of *Anthopleura* COXI sequences. (*C*) Maximum likelihood tree of phased haplotype AnthoYFP sequences; green clade is haplotypes associated with the Neon phenotype; magenta indicates paired sequences from heterozygotes. Node values in trees are ML support based on 1,000 bootstrap replicates; nodes with less than 70% support are reduced to polytomies. (*D*) CIE color analysis of images of genotyped anemones. Each point represents average pixel color of one anemone. Representative images of each genotype are shown below and correspond to the individuals indicated in the CIE color plot.

To determine the genetic basis of the Neon morph, we analyzed the population genetics of Sunburst Anemones at Hopkins Marine Station, Monterey Bay, California (Fig. 2*A*). Intertidal surveys showed that Neon anemones occurred at a frequency of 0.94 ± 0.04% (n = 14 transects, 1,778 sea anemones total). We then collected tentacle clips from replicate pairs of apparent Neon anemones and their nearest wild-type neighbor (n = 14 pairs). No discernable differences in environmental conditions (e.g., depth, light exposure, substrate) were observed within neighbor pairs, as most were separated by <10 cm (maximum distance ≤0.5 m). For comparison, we also sampled the congeners *A. xanthogrammica* and *A. elegantissima* within the same area (n = 3 for each species).

Cryptic speciation occurs in multiple anemone species (37–40), and high levels of genetic divergence exist between color morphs in *Anthopleura* species in the western Pacific (19), suggesting that the Neon morph could represent a speciation event. However, phylogenetic analyses of the Cytochrome Oxidase subunit I gene showed no signature of cryptic speciation between wild-type and Neon morphs, which formed a single clade distinct from other *Anthopleura* spp. (Fig. 2*B*). However, this does not entirely exclude the possibility of population structure that could be observed across more loci. Analysis of the 23S ribosomal RNA gene of the *Symbiodinium* chloroplast showed that all samples harbored zooxanthellae of the same genotype, Clade B subtype IV (*SI Appendix*, Fig. S9), indicating that symbiont genotype does not contribute to coloration differences.

To test whether the AnthoYFP gene correlates with color, we amplified AnthoYFP sequences from each individual and calculated phased haplotypes. Phylogenetic analysis revealed a clade of AnthoYFP haplotype sequences exclusive to Neon individuals (Fig. 2*D*). Most Neon anemones (64%; 9 of 14) were homozygous for this *Neon* allele, while a subset (36%; 5 of 14) appeared to be heterozygous (Fig. 2*C*, pink).

To examine whether *AnthoYFP* zygosity impacts pigmentation phenotype, we quantified coloration in genotyped individuals. Analysis of average tentacle color from images captured during sampling revealed that anemones cluster into three groups that correlated with AnthoYFP genotype: Neon homozygotes clustered in a region of high-saturation green hues, while Neon heterozygotes formed a distinct cluster in an intermediate range of yellow hues closer to the wild-type group, which all fell within an area of low-saturation brown/gray-greenish hues (Fig. 2*D*). Computational analysis of color distances between images using a different, pixel-clustering-based method corroborated this result (*SI Appendix*, Fig. S10).

We next analyzed single-nucleotide polymorphisms (SNPs) between haplotypes (*SI Appendix*, Fig. S11). Most SNPs either encoded synonymous mutations or were unique to individuals. However, one SNP encoding an amino acid shift from a glycine to an aspartic acid residue (G145D) was shared across *Neon* alleles, three non-synonymous mutations were shared in *A. xanthogrammica* (D99N, Y140F, N199K), and one in *A. elegantissima* (L52P). This suggested possible functional differences between AnthoYFP variants, so we isolated and cloned sequences containing these mutations for protein expression and biochemical analysis (*SI Appendix*, Fig. S12) (hereafter abbreviated by species nomenclature as *As*YFP, *Ae*YFP, and *Ax*YFP).

### The *Neon* Allele Is More Highly Expressed and Encodes a Visibly Brighter FP.

**Fig. 3.**
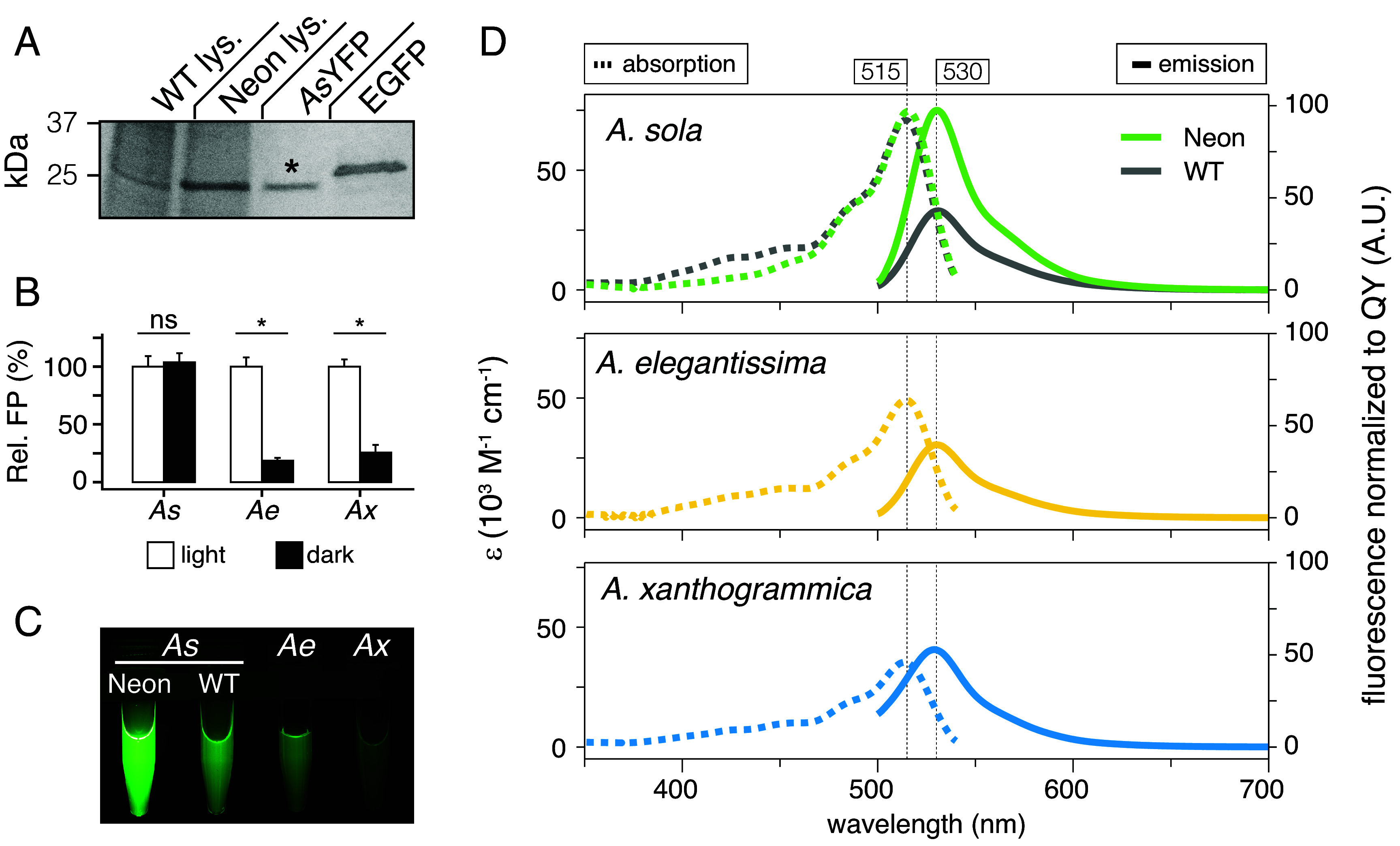
The Neon allele encodes a physically brighter fluorescent protein. (*A*) SDS-PAGE analysis of tentacle lysates from a representative neighbor pair of wild-type (WT) and Neon *A. sola* from genotyping experiments (Fig. 2). Purified Neon *As*YFP protein (asterisk) and EGFP are shown for molecular weight reference. (*B*) Relative AnthoYFP levels from wild-type *A. sola* (*As*)*, A. elegantissima* (*Ae*), and *A. xanthogrammica* (*Ax*) sampled from locations exposed to ambient sunlight (white) or complete darkness (black); values normalized to the average value of the ambient light condition. (*C*) Purified, recombinant FP samples from the three anemone species viewed under ultraviolet light, normalized to 1 mg/mL protein concentration. (*D*) Absorption and emission spectra for the same proteins in *C*; proteins from the two *A. sola* morphs are shown together for comparison (*Top*). Absorption is normalized to the extinction coefficient (Σ), and emission is normalized relative to the quantum yield (QY).

The ~4-fold difference in fluorescence intensity observed between morphs could stem from variation in non-coding sequences around the AnthoYFP gene affecting expression levels or from amino acid differences altering protein structure and chromophore fluorescence. To assess endogenous levels of FP production, we quantified AnthoYFP protein levels in tentacle lysates from genetic sampling pairs and found that the Neon morph produces ~2-fold more AnthoYFP than wild type (Fig. 3*A*, 0.08 vs. 0.03 mg AnthoYFP/mL lysate, normalized to total protein content; *P* = 1.1e^−5^, n = 9, Wilcox test).

However, because levels of FP production in other cnidarians can vary in response to environmental light conditions (18), we tested whether light exposure might explain the observed differences in FP levels in *A. sola*. Comparing lysates from wild-type anemones living in ambient sunlight versus constant darkness, we found no differences in FP levels based on light environment (Fig. 3*B*; *P* = 1, n = 4, Wilcox test; *SI Appendix*, Fig. S3 *B* and *C*). In contrast, both sister species, *A. elegantissima* and *A. xanthogrammica*, showed significant differences in FP levels between light conditions (Fig. 3*B*, *P* = 0.02 and 0.03, respectively, n = 4, Wilcox test).

Given that FP abundance did not fully explain the observed difference in fluorescence intensity between color morphs of *A. sola* (~2-fold vs. ~5-fold), we tested whether the Neon-specific G145D mutation affects molecular brightness. We expressed and purified recombinant AnthoYFP variants in *E. coli* (Fig. 3*C*). Fluorescent spectrophotometry showed that the *Neon* allele encodes a physically brighter FP with a higher extinction coefficient and quantum yield that together predict a theoretical brightness that is more than two times greater than the wild-type allele (Fig. 3 *D*, *Top* and Table 1), which was closer in brightness to the dimmer FPs from *A. elegantissima* and *A. xanthogrammica* (Fig. 3 *D*, *Middle* and *Bottom*).

### AnthoYFPs Function as Antioxidants.

**Fig. 4.**
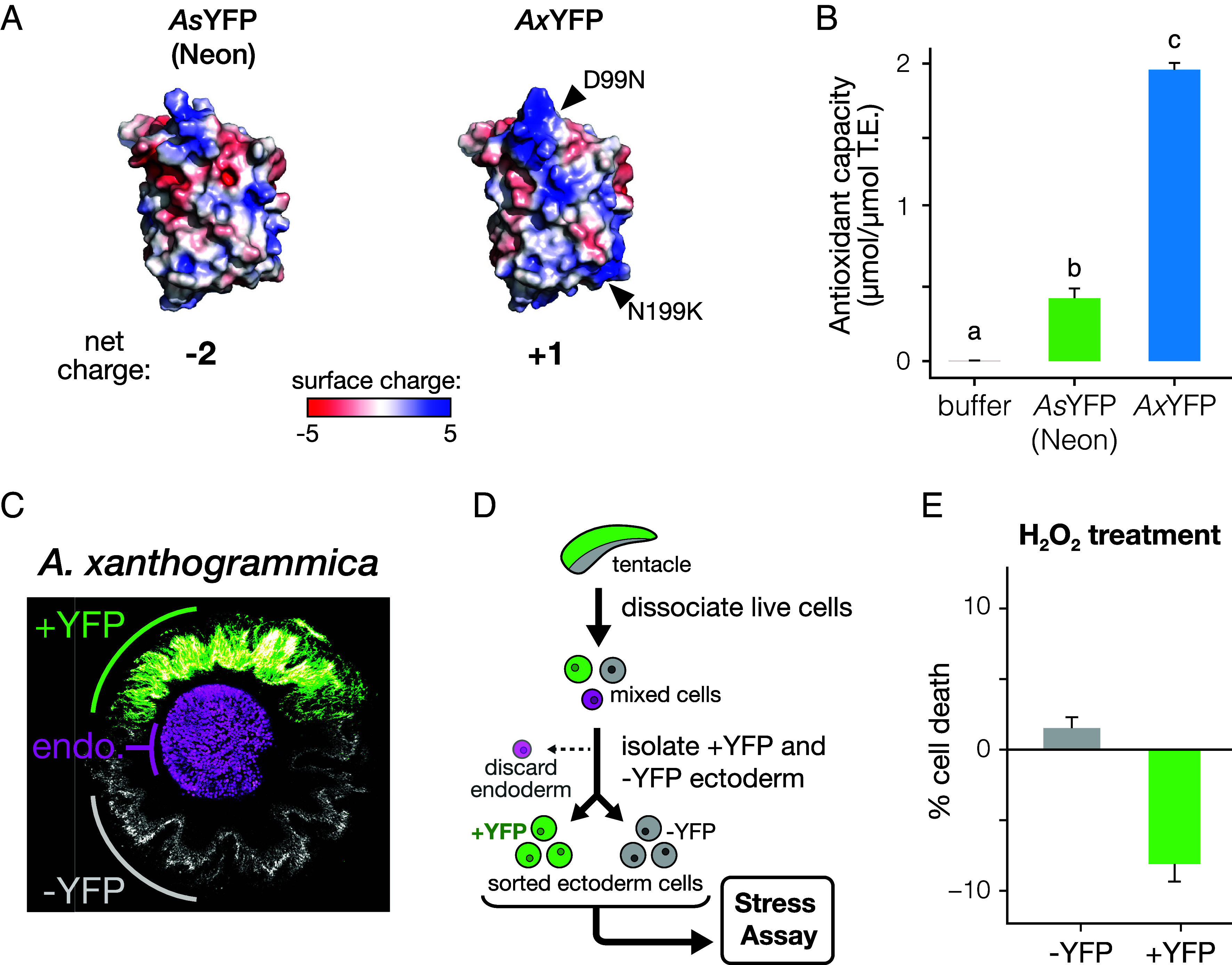
AnthoYFPs can act as antioxidants to inhibit ROS-induced cell death. (*A*) Comparison of surface charge models of AnthoYFPs from Neon *A. sola* (*As*) and *A. xanthogrammica* (*Ax*). Charged amino acids specific to *Ax* are indicated (black arrows). Net charge based on amino acid content is shown below. (*B*) Total antioxidant capacity of purified *As* Neon and *Ax* AnthoYFP proteins. Bars not sharing a common letter are statistically significant from one another based upon a Tukey HSD test (n = 10; statistical bins based on *P*-values < 0.05). Antioxidant capacity is reported as molar equivalent to the reference standard, Trolox (T.E. = Trolox Equivalent). (*C*) Representative cross-section of an *Ax* tentacle stained for DNA with DAPI, oriented with oral, sun-exposed surface at *Top*; grayscale: DAPI in YFP-negative ectoderm; magenta: chlorophyll fluorescence in endoderm; and green: AnthoYFP fluorescence. (*D*) Flowchart of the workflow to isolate +YFP and −YFP ectodermal cell populations used in the stress assay shown in (*E*). (*E*) Effect of YFP content on cell death following treatment with H_2_O_2_ to induce oxidative stress. Cells were analyzed for viability by FACS following treatment. Error bars in *E* indicate SE of two biological replicates.

Based on sequence analysis, we noted differences in protein charge between AnthoYFP variants, with all predicted to be more positively charged than commonly used FPs derived from *A. victoria,* like Enhanced GFP (EGFP) and its engineered yellow variant, EYFP (Fig. 4*A* and *SI Appendix*, Fig. S13). Homology modeling revealed that the mutated residues in *Ax*YFP, D99N and N199K, are surface-exposed and generate local areas of positive surface charge in electrostatic models (Fig. 4*A*). We hypothesized that these surface charge differences could impact antioxidant potential by altering the capacity for electrostatic interactions with charged oxidizing agents. To test this, we examined antioxidant capacity of isolated AnthoYFP variants in vitro by measuring how they inhibit the chromogenic oxidation of ABTS (2,2′-Azino-di-[3-ethylbenz-thiazoline sulfonate]) when reacted with hydrogen peroxide and metmyoglobin. We found that all AnthoYFP variants have statistically significant and functionally relevant antioxidant potential (Fig. 4*B*, Table 1, and *SI Appendix*, Fig. S12). *Ax*YFP was the most potent antioxidant, with ~4- to fivefold stronger activity than the other FP variants, and nearly twofold higher than the reference standard.

To investigate whether the antioxidant capacity of FPs correlates with overall net charge or local surface charge, we compared wild-type *As*YFP to *Ae*YFP, as these FPs have equivalent net charges but differ in surface charge. We found that *Ae*YFP, which has higher surface charge, also exhibits higher antioxidant capacity (*SI Appendix*, Fig. S13 *A* and *B*). We also observed a similar trend between EYFP and EGFP in control experiments (*SI Appendix*, Fig. S13 *C* and *D*), suggesting that this could be a general property of FPs not exclusive to AnthoYFPs.

Based on the strong antioxidant capacity of *Ax*YFP, we focused on this variant to test whether AnthoYFPs have a protective effect against oxidative stress within anemone cells. We dissociated live cells from tentacle samples and used fluorescence-activated cell sorting (FACS) to analyze and later sort ectoderm cells from symbiont-containing endoderm cells (Fig. 4 *C* and *D* and *SI Appendix*, Fig. S14 *A* and *B*). We subjected the isolated ectodermal cell population to physiological stressors, including oxidative stress (H_2_O_2_), UV light exposure, and thermal shock (37 ºC). After treatment, cells were stained with the membrane-impermeable dye DAPI to assay cell viability and analyzed by FACS to assess cell survival and YFP content. For all treatments, the frequency of dead cells was higher in the YFP-negative population than in YFP-positive cells, with the strongest effect observed in the H_2_O_2_ treatment (*SI Appendix*, Fig. S14*C*). To control for the possibility that AnthoYFP fluorescence might be quenched by the treatments, or lost due to cell death, we repeated these experiments using pre-sorted YFP-positive and YFP-negative cells (Fig. 4 *D* and *E* and *SI Appendix*, Fig. S14*D*). Although no relative decrease in cell death was seen in cells exposed to UV and heat shock, pre-sorted YFP-positive cells still displayed increased survival following H_2_O_2_ exposure, confirming that this response was not an artifact of the analysis strategy (Fig. 4*F* and *SI Appendix*, Fig. S14*D*). A similar effect was also observed upon treatment with a different oxidizing agent, tert-butyl hydroperoxide, indicating that AnthoYFP has a general protective effect, regardless of the source of reactive oxygen species (ROS) (*SI Appendix*, Fig. S14*E*).

## Discussion

### Physiological Function of AnthoYFPs.

Questions remain regarding the physiological functions of FPs in intertidal *Anthopleura* species and the extent to which functions are shared between species. Our data demonstrate differing antioxidant capacity across species (Fig. 4*B* and *SI Appendix*, Fig. S13*B*), and species-specific regulation of AnthoYFP production (Fig. 3*B*) (41), suggesting potential divergence in physiological roles. *Ax*YFP is a potent antioxidant, and because FP expression is linked to environmental light exposure, the protein may serve primarily as an antioxidant buffering against ROS stress associated with photosynthesis by algal symbionts and with environmental exposure. In contrast, *As*YFP is encoded by a unique allele with expression more closely related to genotype than to light exposure. *As*YFP is also a weaker antioxidant, but fluoresces more brightly, and so may have a distinct function, such as photoprotection. To clarify this, further studies must link biochemical properties of AnthoYFPs to their physiological functions across species.

### Photobiological Functions of FPs in Endosymbiosis.

In many cnidarians, FPs play diverse roles in regulating algal endosymbiosis, such as providing a spectral cue to attract free-swimming symbionts (17), or modulating the internal light environment to control photosynthetic efficiency (18). Typically, the FPs that regulate photosynthesis are red fluorescent proteins that convert the green light more prevalent in mesophotic habitats into longer wavelength red light (640 to 660 nm) useful for photosynthesis (14). However, AnthoYFP emissions lack the spectral overlap required to excite algal photopigments (*SI Appendix*, Fig. S4). Nonetheless, AnthoYFP appears to be linked to algal symbiosis: the intensity of chlorophyll fluorescence differs between morphs of *A. sola* (*SI Appendix*, Fig. S3*G*) even though they harbor similar numbers of symbiont cells (*SI Appendix*, Fig. S2*E*), which suggests variation in symbiont productivity between morphs. This is consistent with other work linking stable cnidarian symbiosis to ROS stress tolerance (42).

### Sources of Color in *Anthopleura* spp.

The literature commonly suggests that green pigmentation in *Anthopleura* spp. is due to algal endosymbionts (24, 31). However, previous studies on anthozoans, notably *A. elegantissima*, have reported fluorescent green pigments that occur exclusively in the ectoderm, distinct from the symbiont-containing endoderm (41, 43). Our findings corroborate these observations and identify AnthoYFP as the pigment molecule responsible for the green coloration. Still, numerous questions about pigmentation remain, as these anemones display a spectrum of colors—including shades of pink, purple, blue, cyan, dark indigo, opaque white, and red—whose underlying pigments remain unidentified (24). Some evidence suggests that additional chromoprotein and FP genes besides AnthoYFP may contribute to these colors (44, 45), but a comprehensive study of FP and pigmentation diversity is needed to fully answer these questions.

### Regulation of FP Expression.

Our data show that FP production in these sea anemones is regulated by both allele-specific and light-responsive gene expression. In *A. sola*, FP levels depend on the genotype (Fig. 3*A*) and are not strongly affected by the ambient light environment (Fig. 3*B*). The deep-sea anemone *C. japonica* is similar in that FP expression level is also linked to genotype (35), suggesting that allele-specific expression may be widespread in anthozoan cnidarians. In contrast, FP expression in *A. elegantissima* and *A. xanthogrammica* is closely tied to environmental light levels (Fig. 3*B*), as in corals, whose FP production co-varies with sunlight exposure (18, 46).

### Molecular Basis for Brightness Differences between AnthoYFP Variants.

Our findings reveal that the G145D mutation in the Neon AnthoYFP variant drastically increases brightness, but the underlying mechanism is unclear. Alignment of AnthoYFP to the crystal structure of the closely related FP AsFP499 (*SI Appendix*, Fig. S15*A*) (36, 47), and de novo structure prediction with AlphaFold2 (*SI Appendix*, Fig. S15*B*) (48), both place G145D at the start of β-strand 8, ~1.5 nm from the chromophore in a region not known to interact directly with the chromophore in other FPs (2). The only predicted structural effects of G145D are a more open conformation at the distal ends of β-strands 7 and 8 (*SI Appendix*, Fig. S15*C*), and the formation of a salt bridge with the adjacent arginine residue on β-strand 9 (R171; *SI Appendix*, Fig. S13*D*). Both of these molecular features possibly contribute to stabilizing the conformation of β-8 and -9 and increasing chromophore rigidity, which can influence molecular brightness (49).

Alternatively, the G145D mutation may affect brightness through long-distance effects on the conformation of β-strand 8, or interactions between unstructured loops at the β-barrel’s end that alter the position of the internal, chromophore-bearing helix. While the exact mechanism remains unclear, the impact of G145D on brightness is supported by engineered GFP variants with mutations in the same region (50–53), and, remarkably, even in this exact position (54), which enhance brightness and stability (*SI Appendix*, Table S1). Determination of the crystal structures of AnthoYFPs may provide useful insights for designing brighter synthetic FPs.

### Antioxidant Properties of FPs.

In coral FPs and CPs, an inverse correlation has been suggested between fluorescence and antioxidant capacity, with non-fluorescent CPs and weakly fluorescent FPs being more potent antioxidants than brightly fluorescent FPs (13, 15). Our results differ: in AnthoYFPs, antioxidant capacity is not inversely related to molecular brightness. All variants we studied exhibited both bright fluorescence and significant antioxidant capacity (Fig. 4*B*; Table 1). This aligns with prior in vitro studies showing that fluorescent EGFP can quench various forms of ROS, including superoxide and singlet oxygen radicals (55). Moreover, our work shows that single point mutations can enhance both brightness and antioxidant capacity simultaneously, as is seen when comparing the Neon versus wild-type *As*YFP. This suggests that these functions can co-exist within a single protein.

Our results reveal a potential mechanism for FP antioxidant capacity in which positive molecular surface charge enables FPs to interact with negatively charged free radicals. We observed increased antioxidant capacity correlating with mutations that increase local surface charge, potentially linking zones of positively charged surface to antioxidant capacity.

Past studies have suggested that FP antioxidant capacity stems from oxidation of the fluorescent chromophore during its synthesis or decay, and our results do not disprove this model. Analyzing antioxidant capacity in FPs with specific surface charges, such as engineered “super-charged” GFP variants (56), could provide a more direct test of whether surface charge dictates antioxidant potential. Alternative mechanisms, such as surface electrostatics promoting protein stability via increased intramolecular interactions (57), could also boost antioxidant potential by altering the rate of formation of the fluorescent chromophore, or its rate of decay, as both processes include oxidative reactions that depend on interactions between the FP and molecular oxygen (58–60).

We report that an FP’s antioxidant capacity can prevent ROS-induced cell death in cnidarian cells. When considering a potential protective antioxidant function of FPs in cnidarians, it is essential to account for the level of FP synthesis, as the oxidative reactions that generate the FP chromophore also produce free radical O_2_^·–^ and H_2_O_2_, which can trigger an oxidative stress response in cells (61). The three intertidal species of *Anthopleura* studied here, exceptional as sea anemones with algal endosymbionts on the North American Pacific coast (62), are highly adapted to combat excess oxygen radicals produced as byproducts of photosynthesis and UV light exposure (22). Other antioxidants, including catalase and superoxide dismutase (23), are produced in response to excess oxygen from the photosynthetic endosymbionts (63), and mycosporine-like amino acids are synthesized as photo-protectants to block UV irradiation and prevent oxygen radical formation (21, 64). Understanding the dynamics of FP production and antioxidant function in relation to other known antioxidants utilized by sea anemones will ultimately be necessary to comprehend how these animals cope with the extreme stresses of the intertidal environment.

### Genetics and Distribution of the Neon Morph.

Our results show that the *Neon* allele encodes a genetic color polymorphism in *A. sola*, as we observed a stable correlation between the Neon phenotype and *AnthoYFP* genotype that does not vary with environmental conditions or over time. The intermediate phenotype of *Neon*-allele heterozygotes displays an inheritance pattern of incomplete dominance, and approximate codominance, observed with respect to pigmentation.

We detected a north-to-south cline of Neon morph frequencies along the California coast, indicating potential geographic gradient selection (65). This cline may represent a historical pattern or a recent trend in response to changing climate or other habitat impacts. Historical data on distribution of *A. sola* are lacking because, until recently, *A. sola* was considered a solitary morph of *A. elegantissima* (20, 66). However, available data show that *A. sola* is currently undergoing a northward range expansion (*SI Appendix*, Fig. S1) (20, 67), which implies potential for directional selection within populations in central and northern California.

Color polymorphisms can drive speciation in terrestrial animals (68–70) and are associated with genetic divergence in marine invertebrates (19, 71, 72), so the Neon morph could possibly represent an incipient speciation event. Stable polymorphism typically requires balancing selection to maintain approximately equal fitness between morphs (73). Spatial balancing selection, in particular, could provide a plausible mechanism for the maintenance of the Neon morph if it were strongly favored in a rare subset of microhabitats that are more common farther north, with the frequency reflecting the balance between gene flow and selection. In Neon *A. sola*, this could stem from a beneficial role of the Neon AnthoYFP variant in physiology, symbiosis, and/or prey attraction (74). Resolving these questions will require denser sampling and more accurate estimates of allele frequencies across populations, along with a deeper understanding of the various possible functions of AnthoYFPs.

### Possible Fitness Effects of the Neon Morph.

Whether any genotype in the Neon polymorphism has a fitness advantage is unclear. Homozygosity for the *Neon* allele does not have a strong benefit, as the Neon morph occurs at low frequencies, even in localities with relatively higher abundance (e.g., local abundance did not exceed ~15% at any site in our observations). The *Neon* allele could be deleterious because of the metabolic cost of FP over-production, or increased targeting of the conspicuous phenotype by visual predators (11). Although fitness effects of the *Neon* allele cannot be directly inferred from this study, we can speculate based on its geographic distribution. The decreasing abundance of Neon anemones from Monterey Bay southward suggests an interaction between the function of the *Neon* allele and one or more environmental variables that vary along the same range, such as solar irradiance, sea surface temperature, salinity, and pH (75, 76). These variables all decrease with latitude and are therefore inversely correlated with Neon anemone abundance. An intriguing hypothesis for future studies is that the *Neon* allele confers an adaptive advantage to habitat conditions in the northern extent of the range.

## Conclusions

We report a FP gene dictating a color polymorphism and demonstrate antioxidant properties of FPs in vivo. The Neon polymorphism of *A. sola* offers an exciting opportunity to explore phenotypic evolution, given its dynamic distribution and the open questions around fitness and FP function. Our findings suggest a wider role for FPs in cnidarian physiology, notably in managing oxidative stress, and suggest that FPs may simultaneously be under selection for both spectral and physiological functions. The variation in FP properties within this tight clade of *Anthopleura* provides an excellent perspective for probing the evolution of FP functionality.

## Materials and Methods

### Field Observations.

Long-term intertidal monitoring was conducted annually in Pacific Grove, CA, and documented via digital photography. Neon sea anemone abundance at Hopkins Marine Station was estimated by surveying a transect from each identified Neon individual to the nearest average high tide point and counting all Neon and wild-type sea anemones within 1 m of the transect.

### iNaturalist Observations.

A dataset of all research-grade observations of *A. sola* was downloaded from iNaturalist.org (77), and images were screened for the Neon phenotype based on color. Maps were plotted in R using the ggmap package (78, 79).

### Preparation of Tentacle Lysates.

Samples were field collected with dissection scissors into microfuge tubes of chilled seawater and brought into the laboratory within 10 min of excision. Tentacle pieces were weighed and transferred into a 1/20th mass-equivalent-volume (50 µL per 1 mg tissue) of protein isolation buffer (20% glycerol, 1% IPEGAL CA-630, 20 mM Tris pH 8.0, 5 mM EDTA, 1 mM DTT, + 1x Roche Complete protease inhibitor cocktail) and disrupted using a Kontes tissue grinder (Kimble) on wet ice. Samples were further homogenized by 10 passages through a 22-gauge syringe (and sonication with a Sonic Dismembrator (Fisher Scientific) at setting #4 using 10 cycles of 15 s sonication, 45 s resting on ice. Lysates were then centrifuged at 16,000×*g* for 2 h at 4 °C to remove cellular debris and the supernatant collected to a fresh tube. Protein concentration was determined using the BCA protein assay (Pierce BCA Assay Kit, ThermoFisher #23225) and normalized to 1 mg/mL for all lysate samples by addition of buffer.

### Isolation of *As*YFP.

*As*YFP was initially purified from lysates of Neon sea anemone tentacles by ammonium sulfate precipitation and column chromatography. *As*YFP precipitated from tentacle lysate at ~60% saturation with ammonium sulfate. Precipitate was collected by centrifugation, resuspended in 20 mM HEPES, pH 8.0, and further dialyzed against HEPES buffer to remove excess salt. This crude isolate was applied to a MonoS cation exchange column in 20 mM HEPES, pH 8.0, 1 mM DTT. Eluted peak fractions were collected over a 0 to 1 M NaCl gradient. FPs were further purified by size exclusion chromatography (SEC) on a Superdex S200 gel filtration column (Amersham) in 20 mM HEPES, pH 8.0, 150 mM NaCl, and 1 mM DTT. FP-containing fractions were identified by eye and used immediately for further experiments.

### Protein Quantification, Protein Sequencing, Mass Spectrometry Protein ID.

Total lysates and isolated FPs were analyzed via SDS-PAGE. Protein gels were stained with 0.1% Coomassie Brilliant Blue (CBB R-250, 40% methanol, and 1% acetic acid), imaged with a LI-COR scanner, and quantified using ImageJ analysis software. For N-terminal sequencing, isolated FPs were blotted onto PVDF membrane and individual bands were excised and sequenced by Edman degradation. For mass spectrometry protein identification, bands corresponding to FPs were excised from CBB-stained gels and analyzed on a Q Exactive LC–MS/MS system (Thermo Scientific).

### Nucleic Acid Isolation and Molecular Cloning.

DNA and RNA were purified simultaneously using an AllPrep DNA/RNA Mini Kit (Qiagen) according to the manufacturer’s protocol and stored at −20 °C (DNA) or −80 °C (RNA) until used. cDNA libraries were reversed transcribed with the Superscript III First Strand Synthesis Kit (Life Technologies) using oligo(dT) primers. Degenerate primers were designed based on amino acid sequence recovered from mass spectrometry analysis and protein sequencing. PCR reactions for initial YFP gene isolation and molecular cloning were done using Q5 DNA polymerase (New England Biolabs) and inserted into a modified pET protein expression vector (vector 2ST, gift of Scott Gradia, Addgene #29711) to generate in-frame fusion with an N-terminal 6x-His-TEV tag using Gibson assembly (80). Primer sequences used for cloning are provided in *SI Appendix*, Table S3.

### Genetic Analysis.

Tentacle samples were collected from each suspected Neon *A. sola* in the intertidal zone at Hopkins Marine Station, along with its nearest wild-type neighbor (n = 14 pairs). Representative samples from the sister species *A. elegantissima* and *A. xanthogrammica* were also collected (n = 3 each). DNA was isolated as described above. Genotyping PCRs were performed with OneTaq DNA polymerase (New England Biolabs), purified with a QIAquick PCR clean-up kit (Qiagen), and analyzed by Sanger sequencing. For phylogenetic analyses, sequences were first aligned with MUSCLE (81) and trimmed with TrimAl (82). Maximum likelihood trees were inferred using IQ-TREE version 1.6.1 (83): We first used ModelFinder (84) for model selection, and resulting IQ-TREE tree outputs were then bootstrapped with 1,000 replicates using UFBoot2 (85). Resulting trees were visualized in RStudio using the ggtree software package (86). Phased haplotype sequences of *Antho*YFPs were reconstructed using the PHASE software package, version 2.1.1 (87, 88).

### Tentacle Color Analysis.

Brightfield color was assessed using the ColorDistance and Pavo R packages (89, 90). Digital photographs of genotyped anemones were taken at the time of sampling within a single low tide event (within ~2 h) under approximately equivalent ambient light conditions using consistent camera settings between pictures. For color analysis, three sub-sampled RGB images of unobstructed tentacle area were extracted from each photo, and pixel color data was extracted and converted into the CIE-XYZ color space corresponding to human color perception. For plotting, an average pixel color was calculated per individual. For calculating color distance, replicate images were concatenated, and color histograms were calculated for each anemone using default settings. Distances were calculated using the χ^2^ distance metric.

### Recombinant Protein Expression and Purification.

pET expression constructs were transformed into BL21(DE3) *E. coli* (New England Biolabs). Cultures were grown in lysogeny broth supplemented with 100 µg/mL carbenicillin with shaking at 37 °C in baffle flasks, to OD_600nm_ = 0.8. Expression was induced with 1 mM IPTG, and cultures were incubated for 4 h at 30 °C. Cells were harvested by centrifugation, resuspended in binding buffer (20 mM Tris pH 8.0, 500 mM NaCl, 1 mM DTT), and lysed via sonication. FPs were then purified on nickel–nitrilotriacetic acid agarose (Ni–NTA) beads (Qiagen), washed with 10 volumes of wash buffer (binding buffer plus 30 mM imidazole), and incubated overnight with TEV protease at 4 °C in dialysis buffer (20 mM Tris pH 8.0, 200 mM NaCl, 5 mM MgCl_2_, 0.1 mM EDTA, 1 mM DTT) to cleave FPs from Ni-NTA-bound His-tags. The flowthrough was further purified by SEC, and FPs were eluted, normalized to 1 mg/mL concentration in storage buffer (200 mM NaCl, 20 mM Tris pH 8.0, 25% glycerol), and either snap-frozen in liquid nitrogen and stored at −80 °C, or stored on wet ice at 4 °C and used immediately for experiments.

### Spectral Analysis.

Spectra were measured using a SpectraMax i3x spectrophotometer (Molecular Devices) across the wavelength range of 250 to 840 nm with a 5 nm step size. Spectra of storage buffer was recorded as a background reference. Extinction coefficients were calculated following basic denaturation in 0.1 N NaOH (44,000 M^−1^ cm^−1^ at 446 nm) according to the Beer–Lambert law, as previously described (7, 49). Fluorescence quantum yields were measured using fluorescein in 0.1 N NaOH as a reference standard, and EYFP and EGFP expressed and purified under the same conditions were used to calibrate with published values (49).

### Antioxidant Status.

Antioxidant capacity of purified FPs was assessed using the Total Antioxidant Status Assay (Calbiochem Total Antioxidant Status Assay Kit, Sigma Aldrich, #615700). We performed the assay following the manufacturer’s protocol, adapted in scale to be performed in replicates in a multi-well plate within a temperature-controlled spectrophotometer. In brief, 20 µL of either storage buffer, a protein diluted in storage buffer, or the reaction standard (6-Hydroxy-2,5,7,8-tetramethylchroman-2-carboxylic acid; aka Trolox) was added to 1 mL of phosphate buffered saline containing metmyoglobin and ABTS chromogen, and briefly vortexed. 255 µL of each mix was then aliquoted in triplicate into wells of a 96-well plate (Perkin-Elmer #6055630); 50 µL of H_2_O_2_ substrate was then added to each well simultaneously using a multi-channel pipette, and mixed rapidly by pipetting up and down eight times. Reactions were then placed inside of a SpectraMax microplate reader (Molecular Devices) pre-equilibrated to 37 °C. The initial absorbance was recorded and measured again after incubation for 3 min at 37 °C. Samples were analyzed in three replicate reactions in each of five independent experiments (n = 15). Addition of storage buffer alone served as a control reaction, and recombinant EGFP and EYFP proteins served as a reference. All proteins were diluted to 40 µM prior to addition.

### Histology.

Tentacle samples were rinsed in 0.2 µm-filtered sterile artificial seawater (SASW) (91), and then fixed in 4% paraformaldehyde in SASW for 30 min, rinsed with PBS + 0.1% Triton-X100 (PBST), and incubated with Alexa-Fluor-555-conjugated phalloidin (Invitrogen) diluted 1:250 and DAPI (ThermoFisher) diluted 1:10,000 in PBST overnight at 4 °C with gentle agitation. Following staining, samples were thoroughly washed in PBST, and then transitioned through a series of 15% and 30% sucrose in PBS and incubated in OCT embedding medium (Fisher Sci.) overnight at 4 °C. Samples were then snap-frozen on dry ice and sectioned on a cryostat (Leica). Sections were mounted and imaged on a Zeiss LSM-700 scanning confocal microscope.

### Flow Cytometry.

We isolated live cells from freshly collected tentacle samples of *A. xanthogrammica* by mechanical dissociation, following the methods of Rosental (92) and Gates and Muscatine (93): tentacle pieces were rinsed three times in calcium-free artificial seawater (93), minced with a razor blade, further dissociated with gentle trituration in a Pasteur pipette, filtered through a 40 µm mesh, washed, and collected into FACS staining medium (3.3x PBS, 2% fetal bovine serum [FBS; Gibco], and 10 mM HEPES). No enzymatic dissociation was used, and the inclusion of FBS was sufficient to prevent cell aggregation. Cells were stained with DAPI to assess viability and analyzed on a FACS Aria-II instrument (Becton Dickinson) using the following excitation lasers and optical filters (laser wavelength in nm, filters as long pass (LP) and band pass (BP)): DAPI—405 nm (405/50BP), YFP—488 nm (530/30BP), symbiont chlorophyll autofluorescence—640 nm (675/25BP). For sorting, live cells were separated from debris based on intrinsic size (FSC) and granularity (SSC) properties under 488 nm laser excitation, and then gated based on negative DAPI and chlorophyll fluorescence to select live tentacle epidermis cells. This total epidermis population (Fig. 4*C*) was used as input for stress assays on unsorted cells (Fig. 4*E*). For stress assays on sorted cells (Fig. 4*F*), the epidermis population was further gated based on *Antho*YFP fluorescence (as shown in Fig. 4*D*). FACS data were analyzed using FlowJo software, version 10 (FlowJo). For all flow cytometry experiments, a minimum of two biological replicates were performed, and a minimum of 30,000 cells were counted per treatment.

### Cellular Stress Assays.

Live cells (10^5^ cells/200 µL) in FACS staining medium were subjected to thermal stress (30 min at 37 °C), ultraviolet light exposure (10 min under a 254 nm UV-C lamp, 40 µW/cm^2^ irradiance; Labconco), or oxidative stress (1 mM H_2_O_2_ for 1 h at 14 °C), or a control condition (untreated incubation at 14 °C). Cell survival was assessed via FACS analysis, as described above.

### Structural Modeling.

For structural analysis and modeling we used PyMOL version 2.4.0 and AlphaFold2 (48, 94). Surface charges were predicted using the APBS Electrostatics plugin with default settings (95). Structural alignment of AnthoYFP to AsFP499 was done using the Multiple Mapping Method server with PDB structure 29CI, chain E, and the Neon *As*YFP protein sequence as inputs (96). Net charge and theoretical isoelectric point were calculated using the IPC2.0 web server (97), and charge values were verified by manual calculation using polar residues ([#R + #K] – [#D + #E]) (56).

## Supplementary Material

Appendix 01 (PDF)

## Data Availability

All study data are included in the article and/or *SI Appendix*.
