# Supplementary material for: Fluorescent proteins generate a genetic color polymorphism and counteract oxidative stress in intertidal sea anemones
Source: Proc Natl Acad Sci U S A. 2024 Mar 8;121(11):e2317017121. doi: 10.1073/pnas.2317017121 (PMC10945830; doi:10.1073/pnas.2317017121)
Supplement: Supplementary file 1 — Appendix 01 (PDF) [file pnas.2317017121.sapp.pdf]

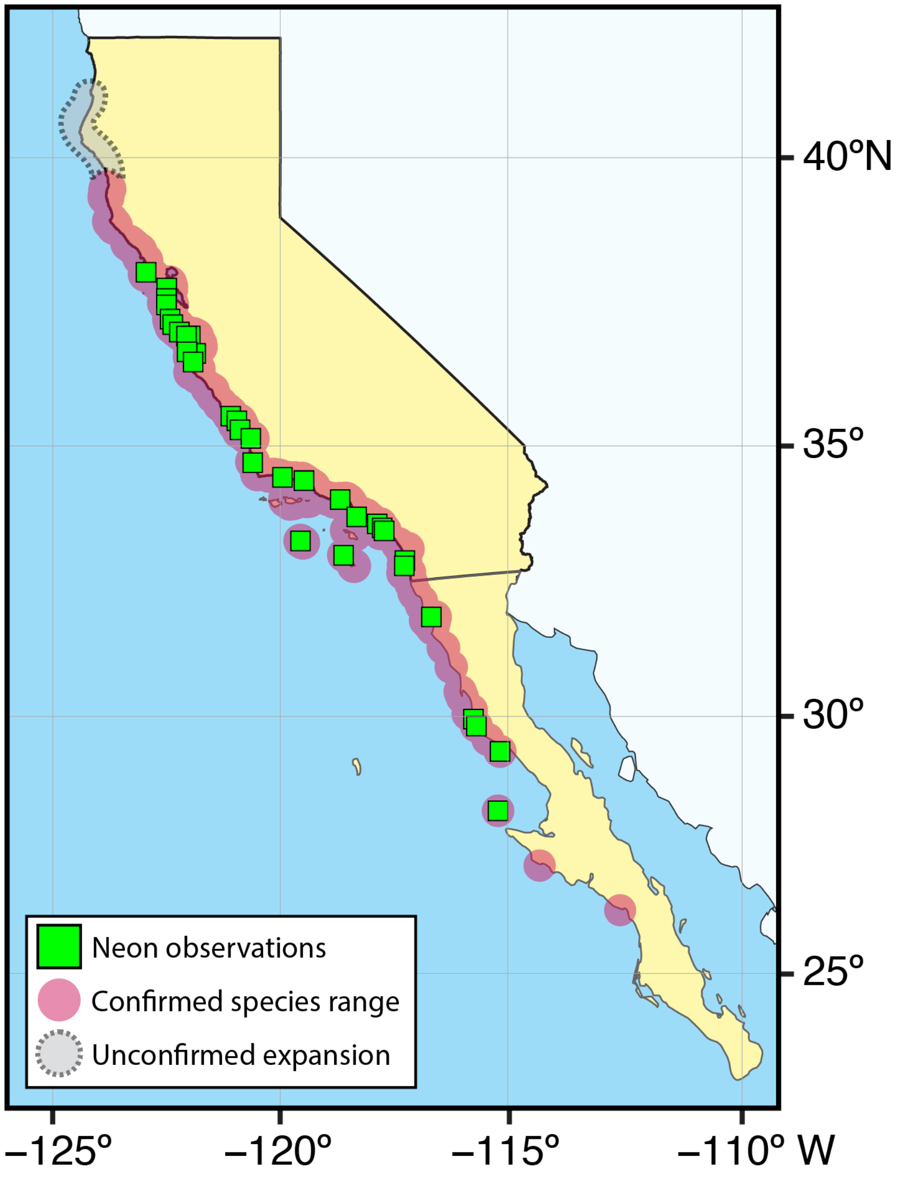

Supplemental Figure 1 – distribution map of *A. sola*. Generated from research grade observations from the iNaturalist database confirmed by historical literature accounts or personal communications with local researchers (magenta). Unconfirmed observations beyond the historical northern extent of the species range are shown in grey. Locations with observations of Neon individuals are shown in green.

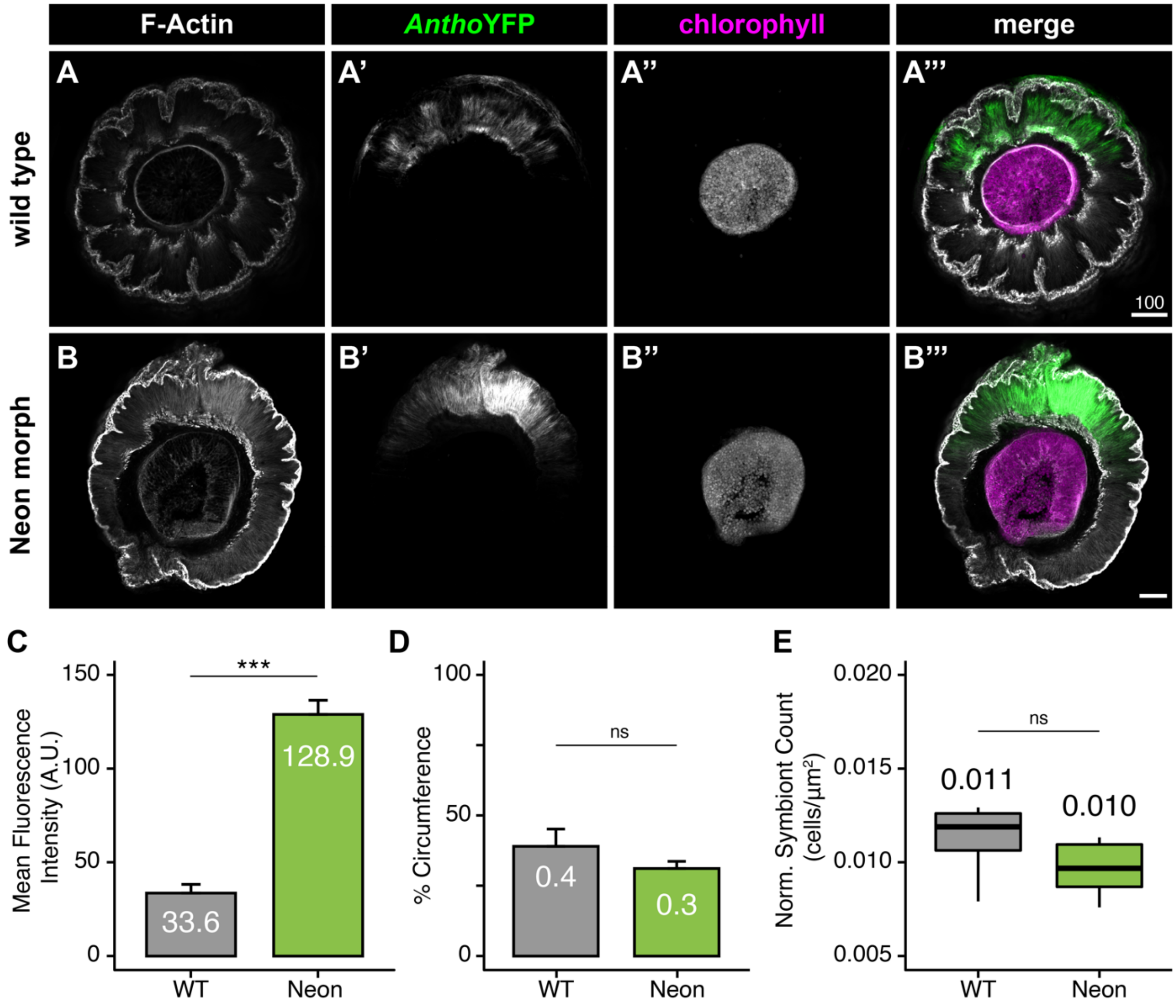

Supplemental Figure 2 – Analysis of tentacle cross-sections of *A. sola* reveal that AnthoYFP is expressed asymmetrically within the ectoderm, and not within the endosymbionts or symbiont-containing endoderm. Asymmetric expression of YFP and symbiont abundance are uniform between color morphs, but total levels of YFP fluorescence are significantly different. Representative confocal micrographs of tentacle cross-sections of wild type **(A-A''')** in comparison to the Neon morph **(B-B''')**; scale bars are 100 μm. **(C-E)** quantifications of the mean YFP fluorescence intensity within the YFP positive region (C), percent circumference of tentacle epidermis containing YFP protein (D), and number of symbiont cells per tentacle area (E); plots are averages of cross-sections from seven individuals of each condition, with error bars showing standard deviation. Differences in (D) and (E) are not significant.

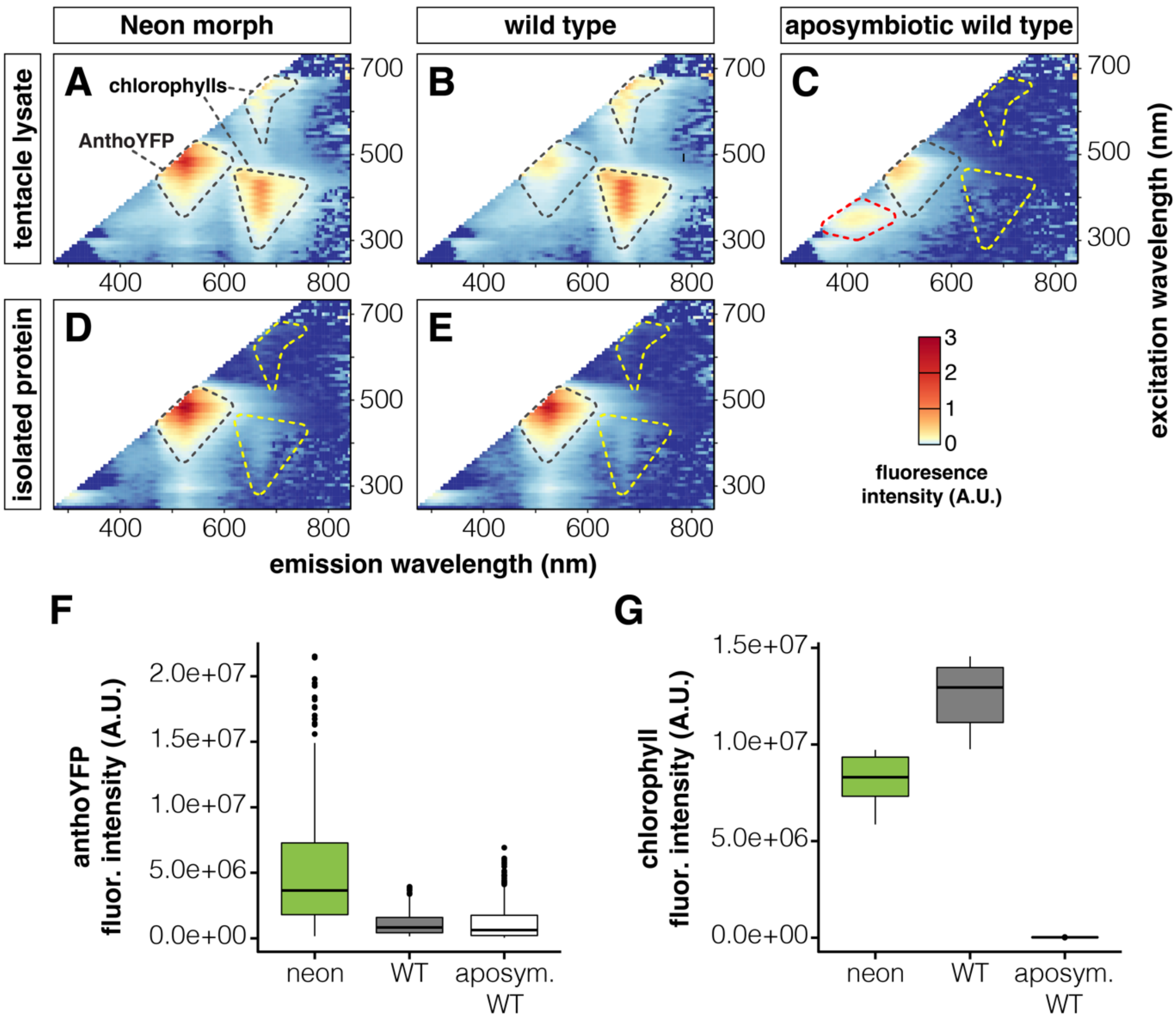

Supplemental Figure 3 – Excitation / emission matrices of tentacle lysates from *A. sola* and isolated proteins. Neon morph (A), wild type (B), and an aposymbiotic wild-type individual containing no algal symbionts (C), in comparison to matrices for isolated fluorescent proteins (D, E). Peaks corresponding to fluorescent protein and algal chlorophylls are indicated (dashed lines). Peak in the ultraviolet spectrum unique to the aposymbiotic condition is indicated in red. **F, G** mean fluorescence intensity of *Antho*YFP (F) and chlorophyll (G) measured from lysate samples collected from aposymbiotic wild-type anemones living in near-darkness, in comparison to wild-type and Neon morph exposed to ambient light in the intertidal. Genotypes of aposymbiotic anemones were confirmed by genotyping PCR.

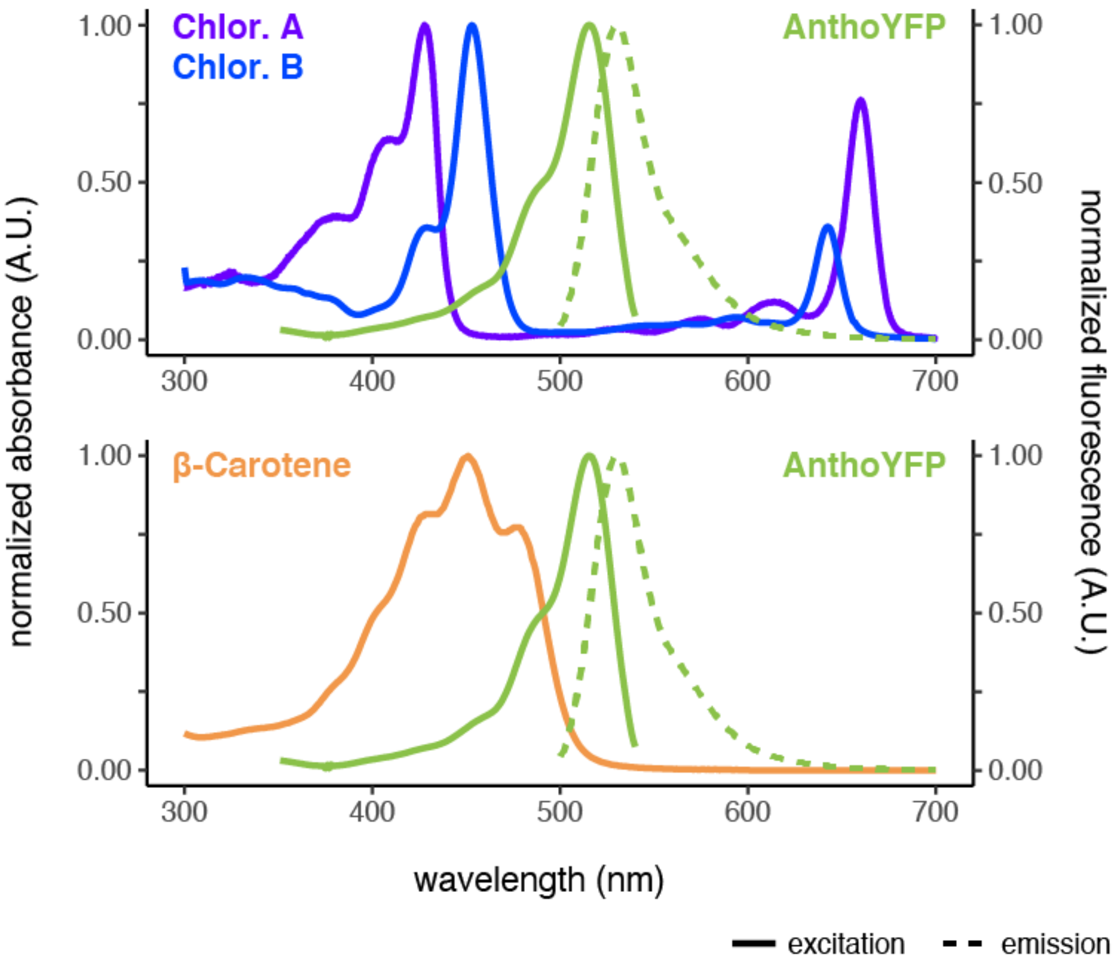

Supplemental Figure 4 - Fluorescent excitation/emission spectra of *Antho*YFP (green) plotted in comparison to excitation spectra for chlorophyll A (purple), chlorophyll B (blue), and β-carotene (orange). Spectra are normalized to peak values.

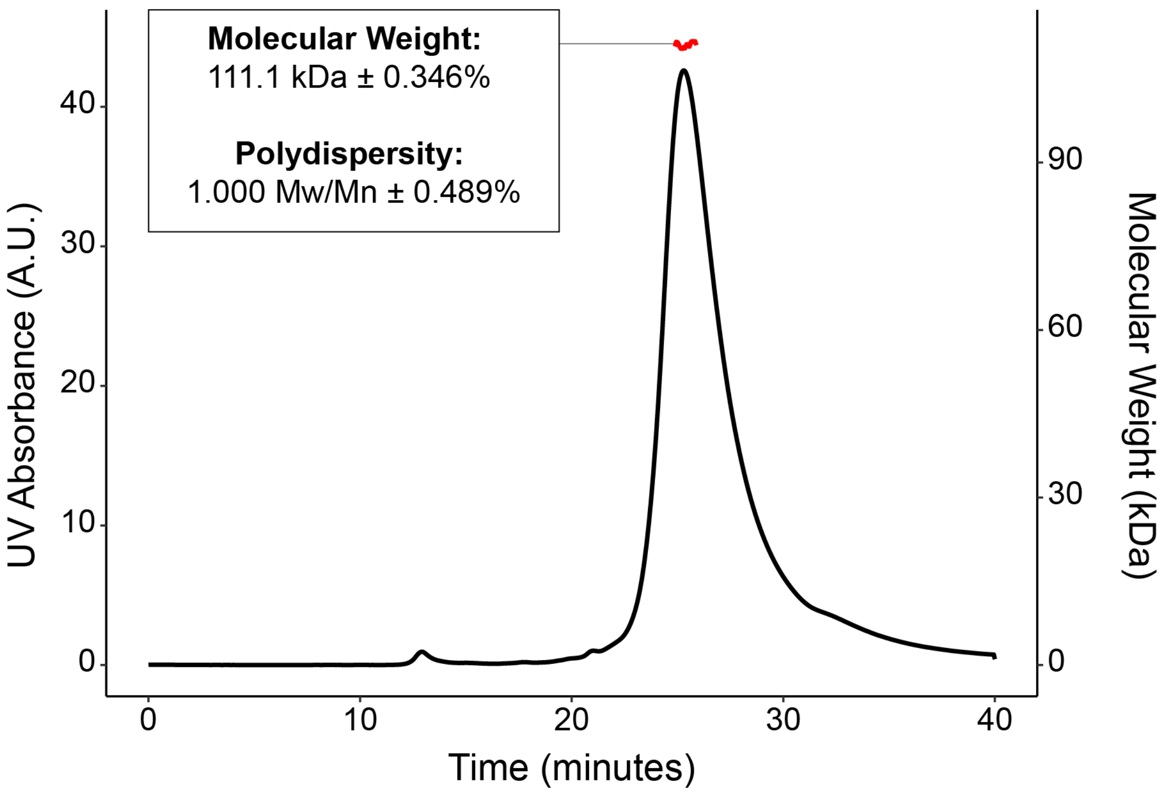

Supplemental Figure 5 – Size exclusion chromatography and multi-angle light scattering analysis of AnthoYFP. AnthoYFP elutes as a single peak with an apparent molecular weight of 111.1 kDa, which is 4 times the predicted mass of the AnthoYFP monomer (27 kDa), indicating a tetrameric state.

AnthoYFP

1 MSGSIKEKMH VKVFMEGSVN YHAFKCTAEG EGNPYEGVHS MKIKVTEGGP LPFAFDILAL CFSYGQKVFI KYPKEIPDFF KQSFPEGYTW ERVTTYEDGG

101 VLSVTQDTSL QGDCLICNVK AIGTNFPPNG PVMKKKTCGW EPSTEIVYPH EGGLIAQDTM ALKLVGGGHL LCFLKTTFRS KKKRITLPEC HFHDYRLETI

201 KESDNGNTID QYEGTVARYS YLPSKLGNH

Coverage	# Peptides matched	MW (kDa)	Exp. q-value	Confidence
62.17%	14	25.734	0.00	HIGH

Supplemental Figure 6 - Summary of mass spectrometry protein identification.

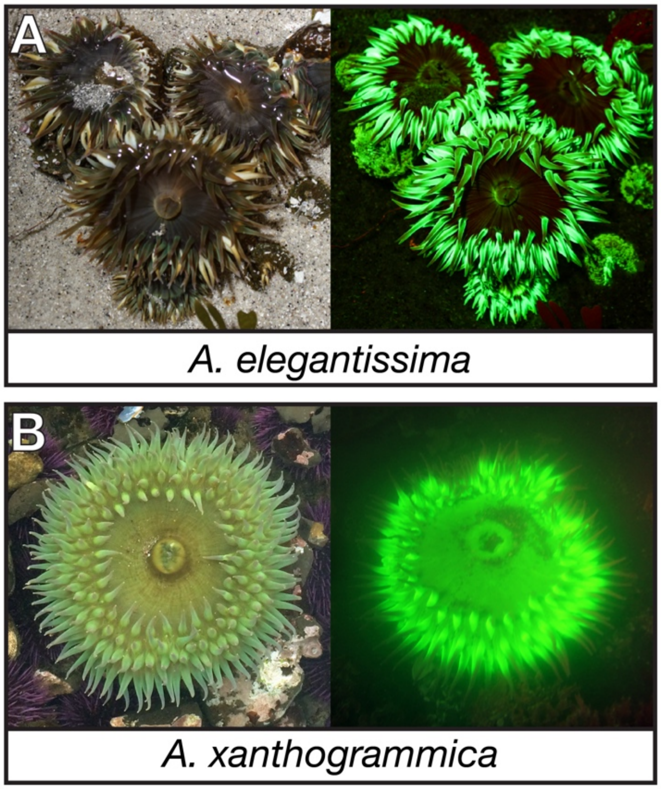

Supplemental Figure 7 – A, B. Images captured under daylight (left) and images captured at night under blue light illumination (right) of *A. elegantissima* (A), and *A. xanthogrammica* (B), demonstrating bright yellow-green fluorescence.

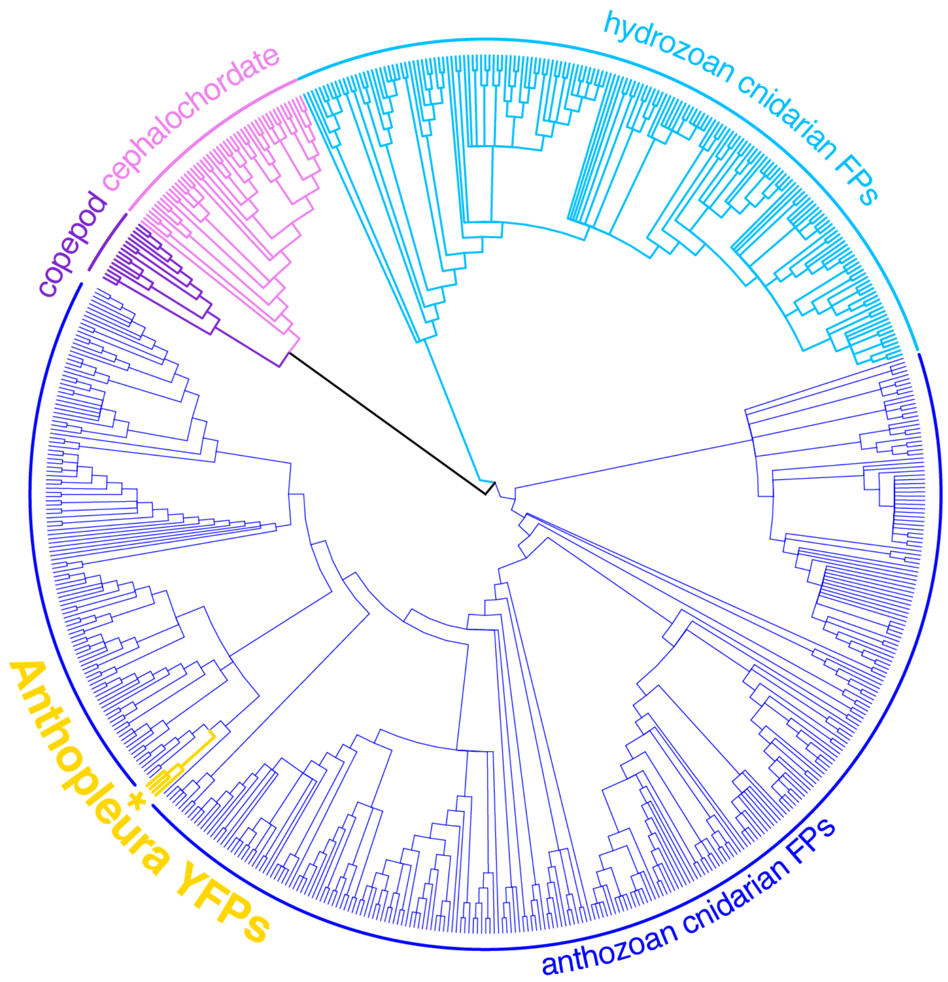

Supplemental Figure 8 - Maximum likelihood phylogeny of metazoan fluorescent protein amino acid sequences (from Yue *et al*. 2016), recalculated with *Anthopleura* YFP sequences (yellow) in relation to other cnidarian FP sequences (anthozoan, dark blue; hydrozoan, light blue) and those from arthropods (purple) and invertebrate chordates (pink). Node with less than 90% support (based on 1,000 bootstrap replicates) have been reduced to polytomies.

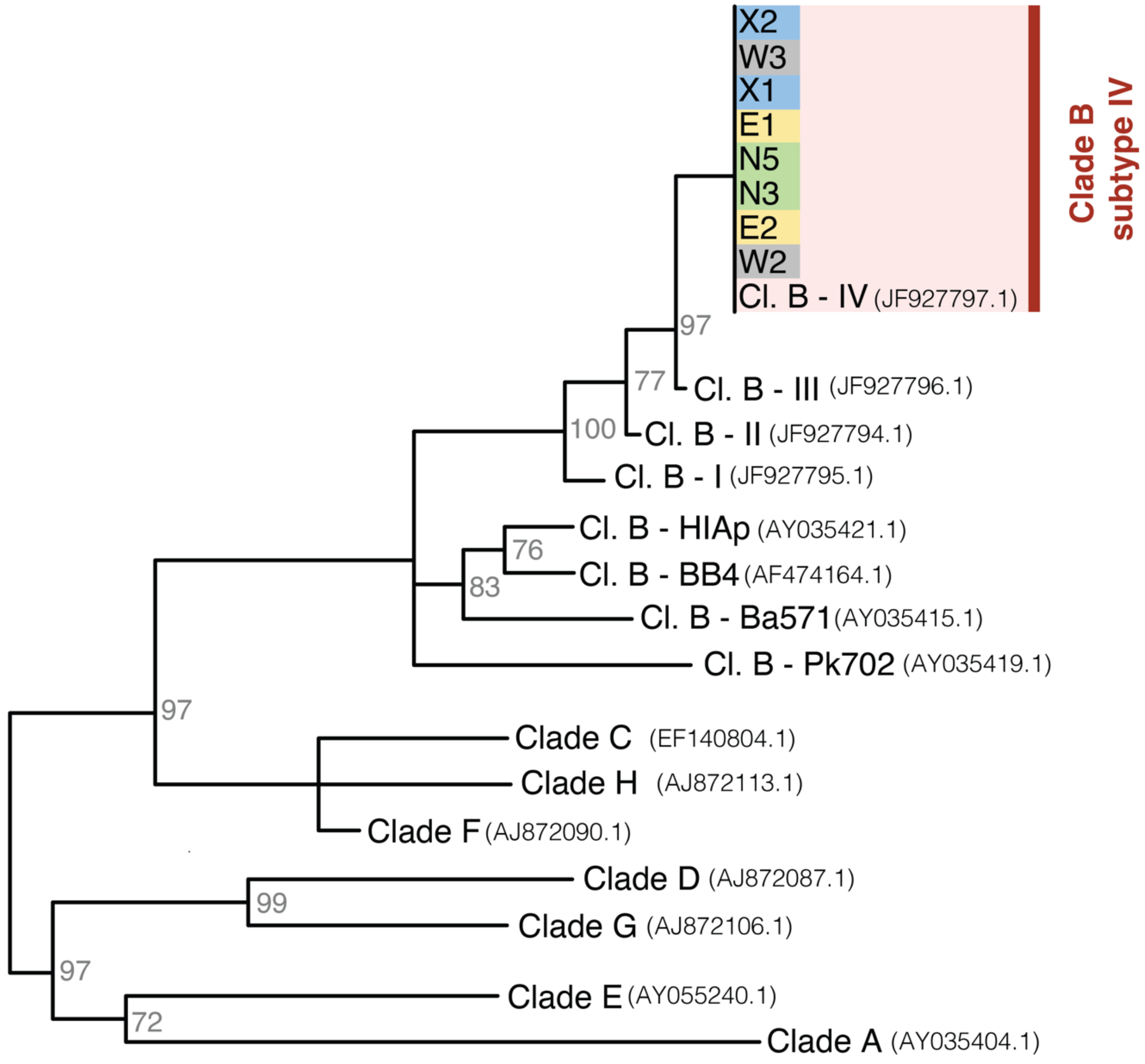

Supplemental Figure 9 - Maximum likelihood tree of Chloroplast 23S gene sequences of *Symbiodinium* sampled from the same individuals as in Figure 2, in comparison to reference sequences. Node values indicate ML support based on 1,000 bootstrap replicates, and nodes with less than 70% support have been reduced to polytomies.

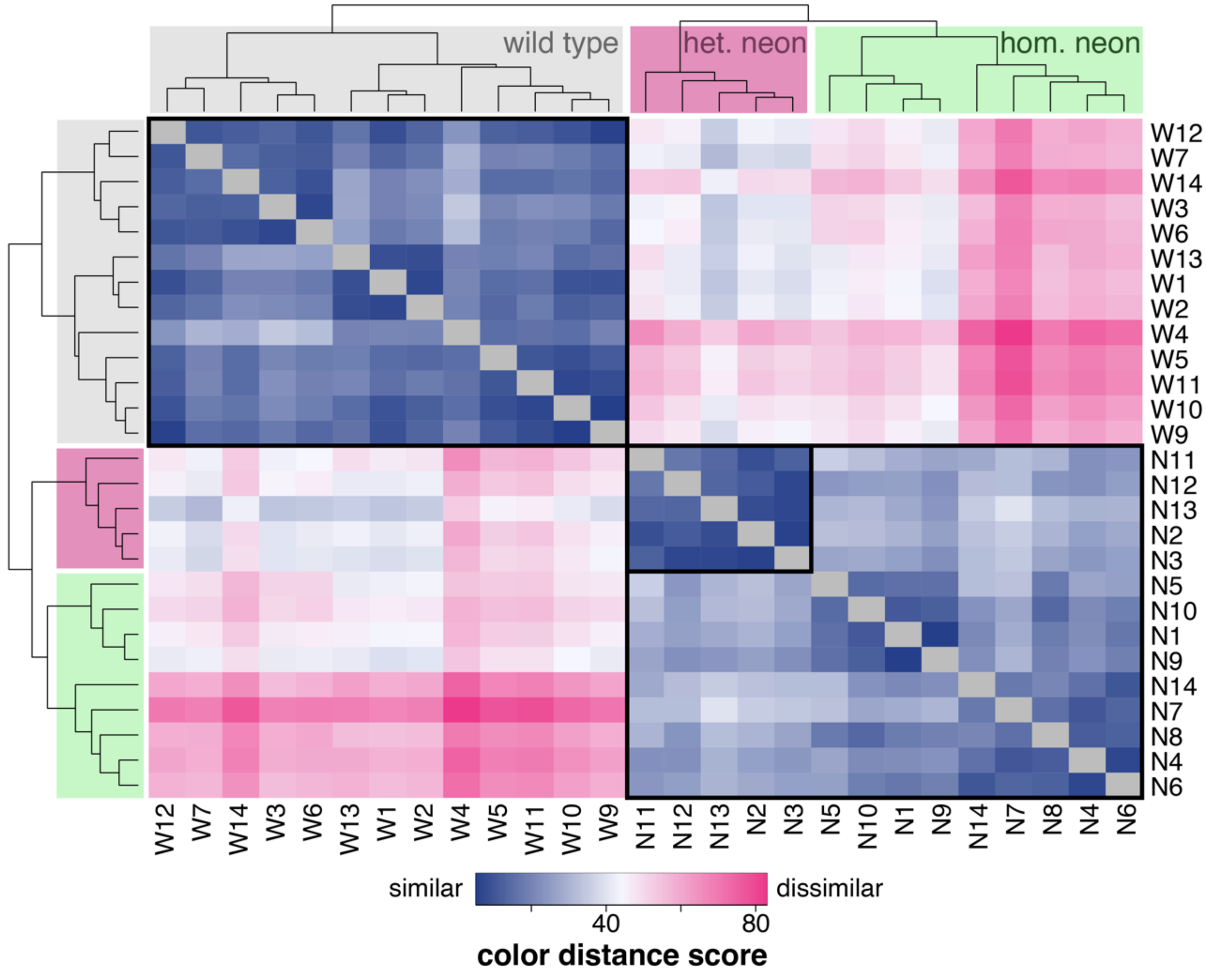

Supplemental Figure 10 - Cluster analysis (color distance) of tentacle coloration computed in the CIElab color space, from the same image dataset of genotyped anemones used for plotting in Fig. 2C. Neon and wild-type individuals cluster independently as two distinct groups, with a sub-group of heterozygous Neon samples forming a sub-cluster with higher similarity to wild-type samples.

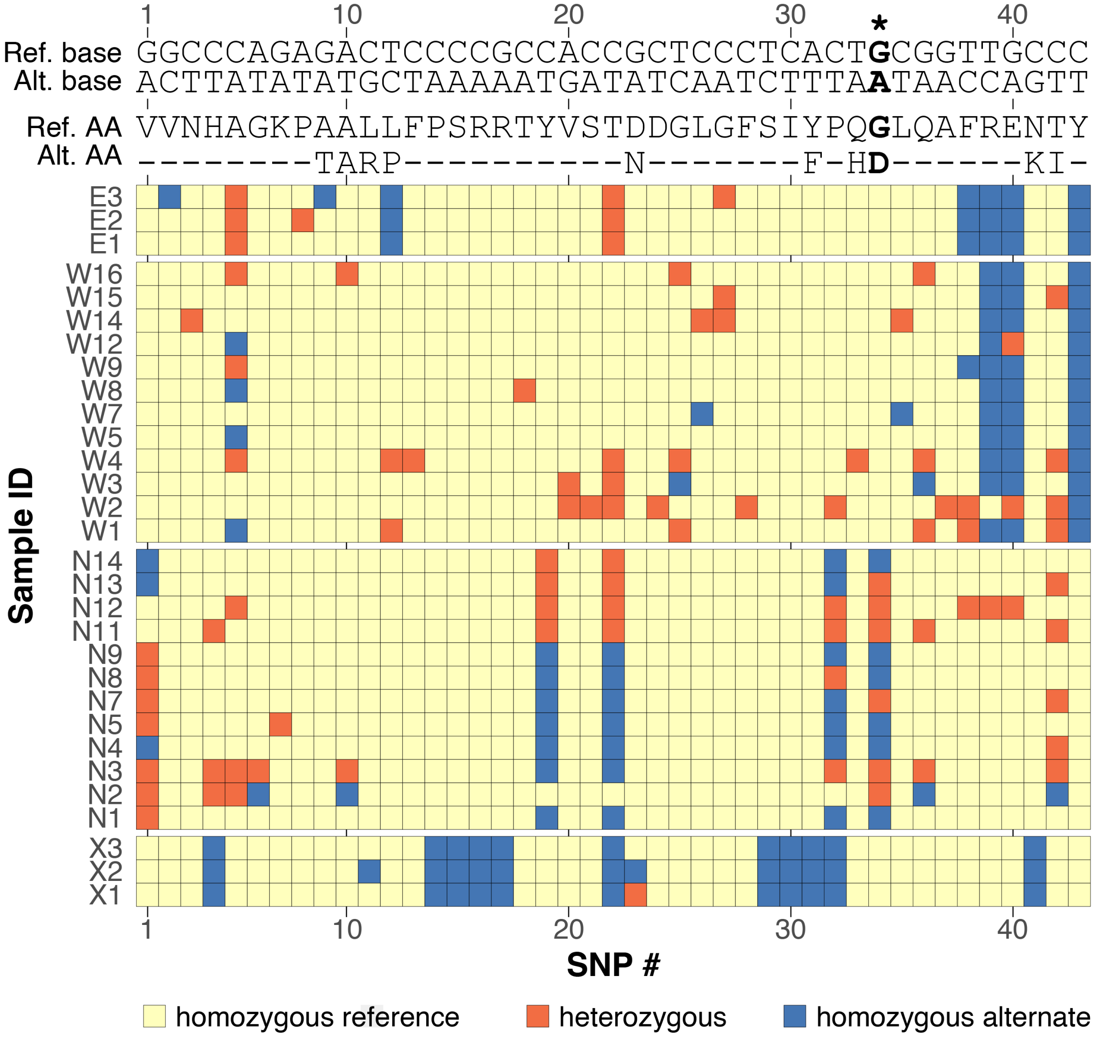

Supplemental Figure 11 - Heatmap of SNPs within the YFP coding sequence for genotyped anemones from the HMS population, corresponding to haplotype sequences in Fig. 2B. Non-synonymous G>D mutation unique to the Neon morph is indicated (asterisk).

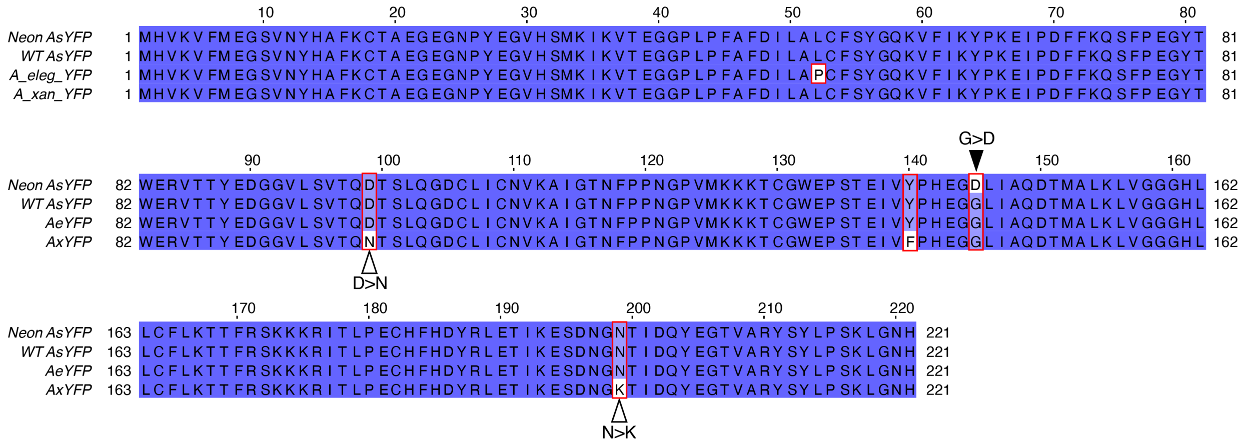

Supplemental Figure 12 - Multiple sequence alignment of amino acid sequences encoded by clones selected for protein expression and purification of *Anthopleura* YFPs. Residues unique to different variants discussed in the main text (Fig. 3 and 4) are highlighted (red); residues specific to the Neon *A. sola* morph (black triangle) and *A. xanthogrammica* (white triangles) are indicated.

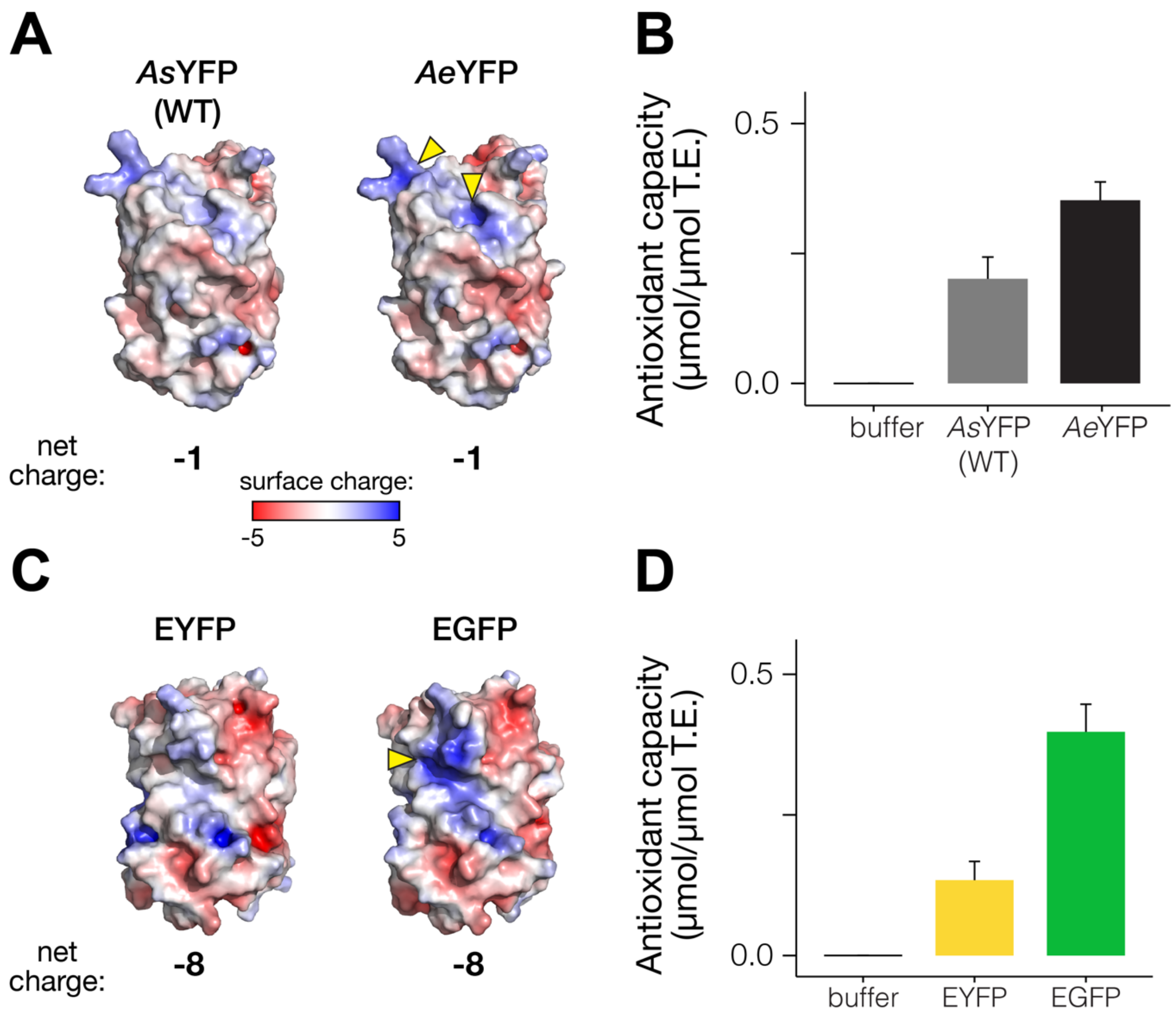

Supplemental Figure 13 – Supplement to Figure 4A-B. **A, B.** APBS surface charge models (A; net charge is shown below) and antioxidant capacity (B) of wild-type *As*YFP and *Ae*YFP. **C, D** same as above, but for EYFP and EGFP.

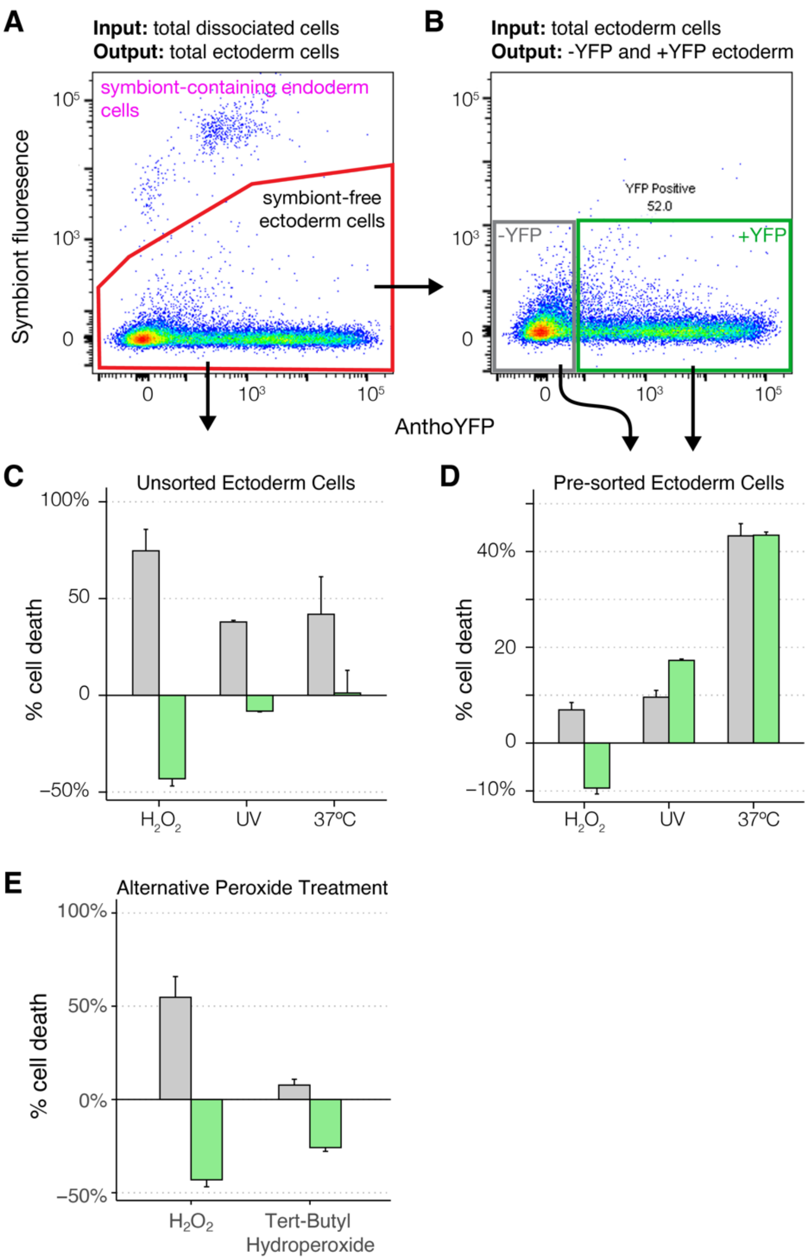

Supplemental Figure 14 – Supplement to Fig. 4D-E. **A, B.** Representative scatter plots demonstrating FACS gating strategy used to isolate symbiont-free cells (A) and YFP-negative and -positive cells (B) from *Ax* tentacle lysates. Arrows indicate cell populations used as input for other experiments. **C** Cell death relative to control for unsorted symbiont-free cells subjected to H_2O_2, UV, and 37ºC treatments. Cells were analyzed for presence/absence of YFP and viability by FACS following treatment. **D** Cell death relative to control for cells pre-sorted based on the presence/absence of YFP as shown in (B), and then treated and analyzed as in (C). **E** A similar protective effect of *Ax*YFP was seen when cells were exposed to the oxidizing agent tert-butyl hydroperoxide (right) as was observed for H_2O_2 (left). Error bars indicate standard error of two biological replicates.

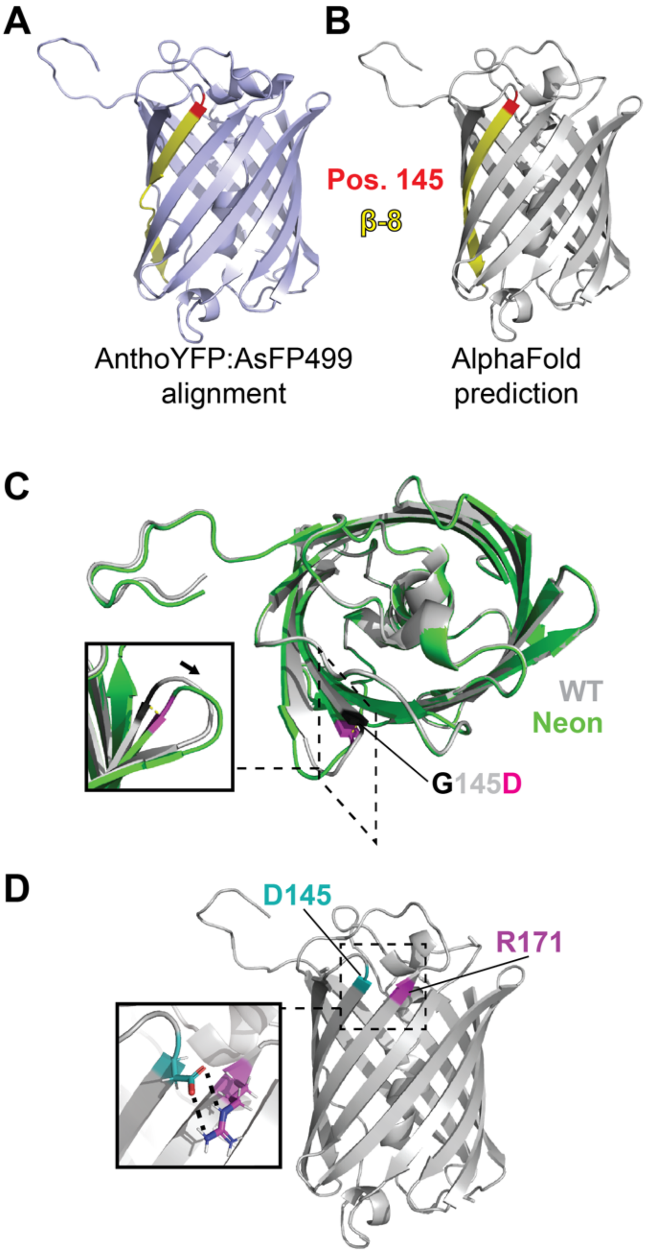

Supplemental Figure 15 – Predicted structure of AnthoYFP and location of the 'Neon' mutation. **A, B** predicted structures of AnthoYFP, generated by (A) alignment of AnthoYFP with the structure of its closest ortholog, AsFP499 (PDB: 2C9I), or (B) as modeled by AlphaFold2; location of the G>D mutation at position 145 is indicated in red, and β-sheet 8 is indicated in yellow. **C** superposition of AlphaFold2-predicted structures of wild-type (gray) and Neon (green) variants, as viewed from the top of the β-barrel; inset shows a side view of the predicted 1.6A outward shift of β-7 and -8 when pos. 149 is mutated from G (black) to D (magenta). **D** proximity of D145 (teal) to R171(purple) at the ends of β-8 and -9; inset shows enlarged view with predicted hydrogen bond interaction between side chains.

FP	Mutation(s)	Relative position	Effect	Ref.
AnthoYFP	G145D	Beginning of β-8 (predicted)	improved brightness	*this study*
mClover	M153T, V163A	End of β-7, mid. β-8	improved brightness, maturation, and stability	28
mVenus				29,30
pH-GFP	M153R	End of β-7	improved brightness	31
mClover3	G160C	Beginning of β-8	improved brightness and stability	32

Supplemental Table 1 – Mutations in lab-derived GFP variants that occur in a similar location the G145D mutation present in the *A. sola* 'neon' YFP. Residue 145 in AnthoYFP corresponds to residue 160 in eGFP and its derivatives.

Supp. Table 2: Sequences of primers used in this study.

Primer ID	Target	Sequence	Purpose	Ref.
AnYFP_deg_F	AnYFP	ATGWSNGGNWSNATHAARGARAARATGC	Initial CDS isolation	This study
AnYFP_deg_R		RTGRTTNCCNARYTTNSWNGGNARR		
AnYFP_F		ATGTCAGGATCAATCAAAGAGAAGATGC	Genetic analysis	
AnYFP_R		CTAATGATTTCCTAGCTTGGAGGGC		
LCO1490	COI	GGTCAACAAATCATAAAGATATTGG		Folmer et al., 1994
HCO2198		TAAACTTCAGGGTGACCAAAAAATCA		
23S1	Chlor. 23S	GGCTGTAACTATAACGGTCC		Zhang et al., 2000
23S2		CCATCGTATTGAACCCAGC		
AnYFP_GA_F	AnYFP	atgaaatcttctcaccatcaccatcaccatATGTCAGGATCAATCAAAGAGAAGATGC	Cloning for protein expression	This study
AnYFP_GA_R		gcgatcgcggatccgttatccacttccaatCTAATGATTTCCTAGCTTGGAGGGC		
pET_F	Exp. vector	ATTGGAAGTGGATAACGG		
pET_His6_R		ATGGTGATGGTGATGGT		
